# Modelling HIV/AIDS epidemiological complexity: A scoping review of Agent-Based Models and their application

**DOI:** 10.1371/journal.pone.0297247

**Published:** 2024-02-02

**Authors:** Rodrigo Volmir Anderle, Robson Bruniera de Oliveira, Felipe Alves Rubio, James Macinko, Ines Dourado, Davide Rasella

**Affiliations:** 1 Institute of Collective Health, Federal University of Bahia (UFBA), Salvador, Brazil; 2 Departments of Health Policy and Management and Community Health Sciences, UCLA Fielding School of Public Health, Los Angeles, California, United States of America; 3 ISGlobal, Hospital Clínic—Universitat de Barcelona, Barcelona, Spain; University of Arkansas for Medical Sciences, UNITED STATES

## Abstract

**Objective:**

To end the AIDS epidemic by 2030, despite the increasing poverty and inequalities, policies should be designed to deal with population heterogeneity and environmental changes. Bottom-up designs, such as the Agent-Based Model (ABM), can model these features, dealing with such complexity. HIV/AIDS has a complex dynamic of structural factors, risk behaviors, biomedical characteristics and interventions. All embedded in unequal, stigmatized and heterogeneous social structure. To understand how ABMs can model this complexity, we performed a scoping review of HIV applications, highlighting their potentialities.

**Methods:**

We searched on PubMed, Web of Science, and Scopus repositories following the PRISMA extension for scoping reviews. Our inclusion criteria were HIV/AIDS studies with an ABM application. We identified the main articles using a local co-citation analysis and categorized the overall literature aims, (sub)populations, regions, and if the papers declared the use of ODD protocol and limitations.

**Results:**

We found 154 articles. We identified eleven main papers, and discussed them using the overall category results. Most studies model Transmission Dynamics (37/154), about Men who have sex with Men (MSM) (41/154), or individuals living in the US or South Africa (84/154). Recent studies applied ABM to model PrEP interventions (17/154) and Racial Disparities (12/154). Only six papers declared the use of ODD Protocol (6/154), and 34/154 didn’t mention the study limitations.

**Conclusions:**

While ABM is among the most sophisticated techniques available to model HIV/AIDS complexity. Their applications are still restricted to some realities. However, researchers are challenged to think about social structure due model characteristics, the inclusion of these features is still restricted to case-specific. Data and computational power availability can enhance this feature providing insightful results.

## Introduction

After 40 years of the HIV/AIDS pandemic, two patterns are currently co-existing: one concentrated in key populations, such as men who have sex with men (MSM), or people who inject drugs (PWID); and the other to heterosexual population, mainly in Sub-Saharan African countries [1]. Both characterized by complex dynamics of infection where Social Determinants of Health (SDH) have an important role on linkage to care (LTC) services [2–4] and transmission dynamics. The complexity inherent to the social structure, subpopulation stigma, and inequality in access to health services, challenges the UNAIDS Fast-Track strategy to end the AIDS epidemic [5]. With the outbreak of Covid-19, the disparities became evident and the UNAIDS once again highlighted the urge for action against inequalities to end the AIDS pandemic [6, 7].

The HIV/AIDS dynamics is accentuate by the complexity of modern social structures. Heterogeneous interactions, combined interventions, and flexibility to different environments claim for suitable approaches and a systemic view of the phenomena [8]. One example is the increase of interactions due the urbanization process [9]. With larger networks, the exposure for unprotected anal intercourse (UAI) increases, among others behaviors [10, 11]. Random interactions are not real. They are embedded in social structure. As the Dahlgren and Whitehead model for SDH shows, individuals have several layers of social conditions affecting their health status [12].

Ethnic minorities can carry a disproportionate HIV burden [13, 14]. Untreated sexually transmitted infections (STI), and fails in linkage to care are some drivers [15, 16]. In addition, the disease burden can be concentrated on transversal groups. PWID, for example, can connect different subpopulations [17, 18]. Additionally, growing inequalities due to economic or humanitarian crises (*e*.*g*. Covid-19, local conflits), hit countries and vulnerable groups differently [19–21] affecting aspects of HIV/AIDS dynamic.

These interrelated multilevel dimensions invoke the use of new approaches [22]. While public health is an inherently complex field that demands holistic methods to work with idiosyncratic individuals [23]. An agent-based Model (ABM) is proposed to simulate the interactions of heterogeneous individuals (agents), understanding insights into these dynamics by modelling non-linear relations [24]. As part of systems science [23], ABMs are a simulation method diffused after the XXI century. Despite some criticism [25], different disciplines applies this method: Economics, Environmental Sciences, Archaeology, and other fields beyond epidemiology [26]. Also identified as Individual Based Models (IBM), the method reproduces a phenomenon by its minor parts, the agents/individuals. Representing heterogeneous agents, interactions, subpopulations, and multiple interventions in different environments. ABMs are flexible to deal with the HIV/AIDS challenges, including its SDH dimension. In fact, as an individual-based method, researchers are obeyed (should be) to think about SDH.

We found some reviews about ABM application on different disciplines [25–27]. Applications on public health [23, 28–30]. And some applications on noncommunicable diseases [31] and malaria [32]. However, no one review the ABM applications on HIV/AIDS-related field. Therefore, our aim is to perform a scoping review of all ABM applied studies to simulate HIV/AIDS dynamics, identify them, collect and systematize their aims, population, regions represented, and their declaration of limitations and protocol use. Moreover, our study aimed to provide a big picture of ABM applications related to HIV/AIDS. We expect this study work as a starting point for researchers and policymakers about ABM applications on HIV/AIDS.

## Methods

### Search

We followed the PRISMA extension for Scoping Reviews [33] to identify studies and assess the ABM uses in HIV/AIDS-related research. We used three independent databases to perform the literature search: 1. PubMed/Medline 2. Web of Science and 3. Scopus. We restricted the search to articles published before May 17, 2023. To obtain target articles, we used Medical Subject Heading (MeSH) and non-MeSH terms with the keywords “HIV” or the term “human immunodeficiency virus”, in the title. And the terms “Agent-Based”, or “Agent Based”, or “Individual-Based”, or “Individual Based” in any part of the paper. A detailed description is in S1 Appendix.

We restrict our search to ABM and IBM understanding they represent a specific topic in simulation models related to complex systems [26]. However, the literature is consensual about this. Some can understand the terminology as interchangeable also with Cellular Automata (CA), Network Models, microsimulation, and others [25].

We defined two inclusion criteria for the studies:1) they should be focused on epidemiological aspects of HIV/AIDS and 2) they should apply Agent-Based Models. Studies without epidemiological applications of HIV/AIDS or without Agent-Based Models applications were excluded. The titles and abstracts of articles were screened to meet the inclusion criteria. Two researchers independently conducted this check and discussed their divergence.

### Main papers

**Fig 1:**
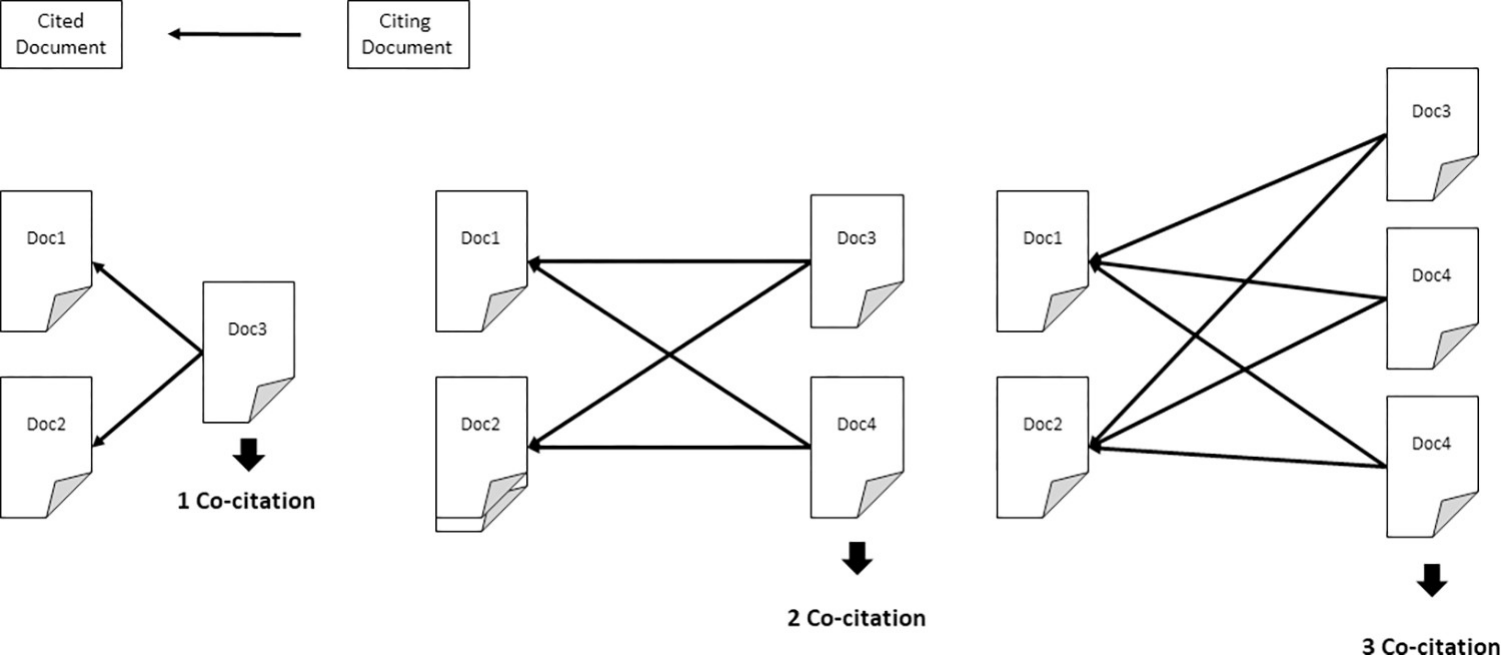
Representation of the co-citation measure.

With the set of papers defined, we performed a local co-citation (LcoCi) analysis to identify the main studies in the field, recognizing foundational themes in the literature [34, 35]. This analysis is a bibliometric tool that explores bibliographic data as a source of information on how knowledge is constituted and diffused. One advantage is the main papers were selected from the literature itself (with its inner bias). A co-citation means they are acknowledged by the field and occurs when two papers are cited by a third paper (see Fig 1) which is different from global citations (GCs) that reflect acknowledgment beyond the field. A local co-citation (LcoCi) means that we considered just the co-citations between the set of papers we collected. We developed this part of the analysis using the Bibliometrix package [36] in the R software. To avoid bias from study-groups we selected every paper with at least four LcoCi.

We also restrict our selection to papers with at least two local citations (LCs). Local citations are computed as the number of citations from the set of papers we collected. A complete list of these results can be found in Table A of S1 Appendix.

### Overall categorization

We also categorized the overall articles based on:1) the aim of the study, 2) its population, 3) its location, and 4) protocol and limitations. Table 1 presents the classification criteria for the “aim” categories.

**Table 1:** Description of the aim categorization.

Aim	Description
ART	Studies focused on antiretrovirals.
Cost-Effectiveness	Studies aimed to evaluate the cost-effectiveness of interventions.
LTC	When the study is focused on Linkage to Care (LTC) interventions or dynamics.
Multidisease	Studies about co-infections of other diseases.
PrEP	Studies focused on PREP interventions and outcomes
Prevention Packages	Studies with more than one intervention (90-90-90, ART, ART; behavioral interventions) and/or their combination on HIV/AIDS outcomes
Proof of Concept	Study that explores some concept or hypothesis by a simulation exercise.
RCT Design	Studies that applied ABM for the design or discussion of Random Control Trials.
Replication Exercise	Replicates a previous application, or a modelling exercise.
Single Intervention	When the study is focused on the effects of only one intervention. Excepting ART and PrEP.
Social Impact	Studies aimed the social impacts of HIV/AIDS
Transmission Dynamic	Study focused on some feature of the transmission dynamic of HIV/AIDS. *e*.*g*. epidemiological burning in specific populations such as MSM or Black/African-Americans.
Epidemiologic Analysis	Studies aimed to predict or analyze some epidemiological measure, such as incidence, or prevalence of HIV.
COVID-19	Studies with a background or interest on the COVID-19 pandemic.

We identified the population of interest in accordance with the article or model description. Regarding location, we identified the studied region and added two more variables: geographic dimension and country. The first describes whether the study is focused: city, state, country… The second identifies the country of this location. This variable is not applicable to global or macroregional studies. We identified each study using one or more categories.

At last, we verified which papers declare the use of ODD protocol [37, 38] and which papers declare the model limitations. The ODD protocol is a protocol developed for better comprehensibility of ABMs. To find its mention, we verify if the paper cites the ODD references [37, 38], or if it mention the term “protocol” on the text (verifying if it is related to the ODD). To access the limitations we searched in each paper the word “limitation” identifying a section or a paragraph addressing the model or paper limitations. We synthesized this information into tables and figures, providing a descriptive map of the field. All these procedures are detailed in S2 Appendix.

## Results

**Fig 2:**
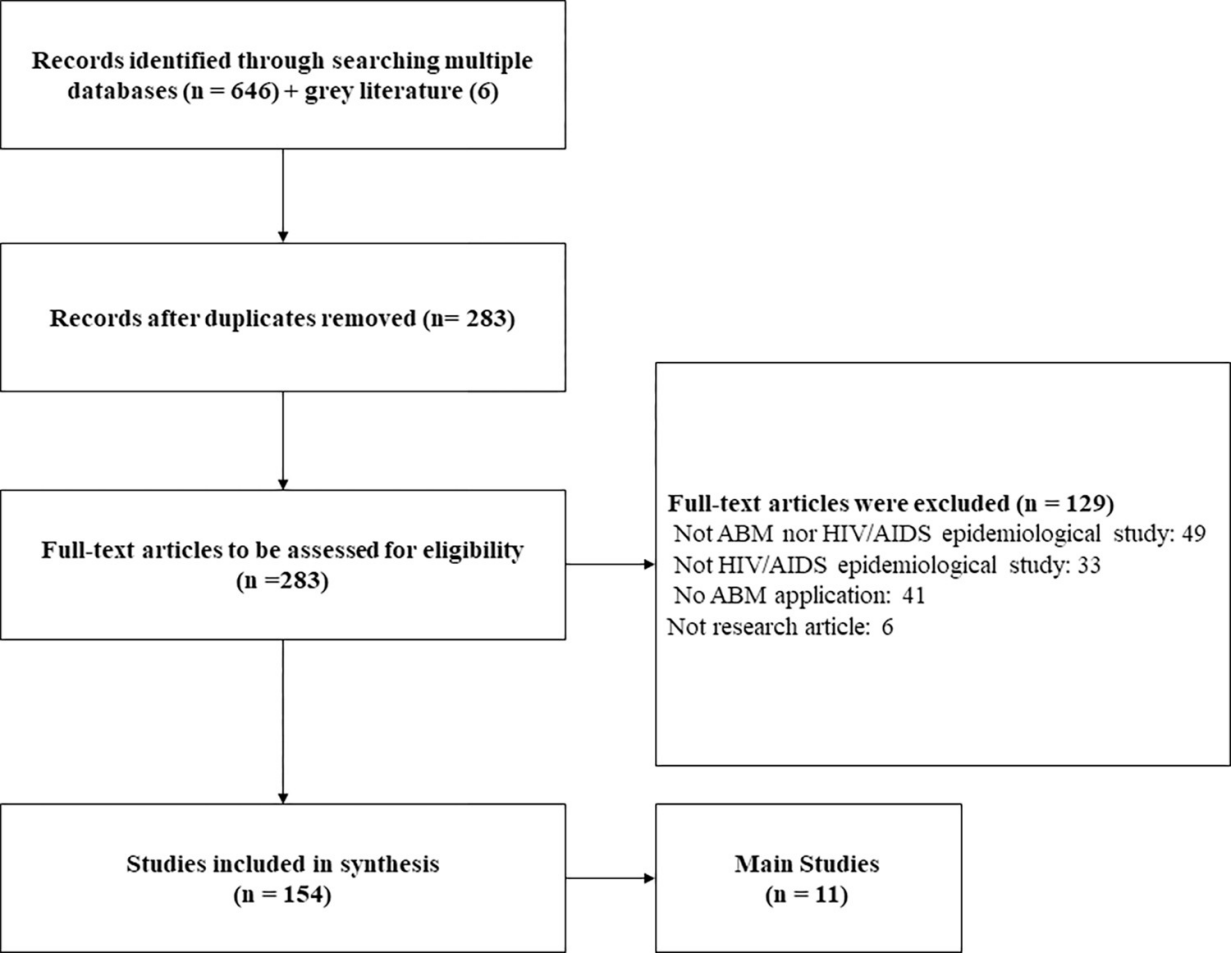
Flow Diagram for selection of evidence sources.

Fig 2 shows the search results. Our search returned 652 articles: 197 from PubMed, 198 from Web of Science, and 257 from Scopus. Deduplicating these findings yielded 283 articles. 154 met our inclusion criteria. We excluded 129 due to: microbiologic studies without relation to ABM (49/129); microbiologic studies without epidemiological goals (33/129); 41/129 studies without ABM application; and six were not published research articles.

The final dataset included 154 articles published between 1999 and 2023. The complete list is presented in S1 Table. 67 different journals published them. Mostly interdisciplinary journals, public health journals, or topic specific journals related to HIV/AIDS. More than half of the studies were published after 2018 (85/154), 45 of them after 2021 (Table A in S3 Appendix).

### Main studies

Table 2 presents the main studies ordered by publication year. Beyrer et al. [39]–who performed the only global study of the selection–and Marshall et al. [40], are the earlier main studies. Beyrer et al. [39] is the most cited article in the selection (more than a thousand citations). They were able to demonstrate the epidemic burden on MSM populations. By modelling the risk increase of unprotected anal intercourse (UAI) among MSM population, and the lower search for health assistance in this population (due to social stigma). They demonstrated how the epidemic trends to concentrated on this population. Marshall et al. [40] used a different approach, focused on a lower geographic dimension, the micro-region of New York metropolitan area. They explore how combined interventions could reduce the incidence of HIV among drug users. The study also explored the trends in subpopulations such as MSM, that varied when considered drug use status.

**Table 2:** The main studies that applied an Agent-Based Model to HIV epidemic identified by co-citation analysis.

Study	Aim	Population	Location	LcoCi	LCs	GCs
Marshall Bdl et al., 2012, Plos One [40]	Prevention Packages	Heterosexual; MSM; WSW; PWID; NIDU; NU	NY Metropolitan Statistical Area (MSA), NY—US	11	7	34
Beyrer C et al., 2012, Lancet [39]	Transmission Dynamics	MSM	Global	4	10	1056
Mccreesh N et al., 2012, Sex Transm Dis [41]	Single Intervention	Representative	Uganda	4	4	23
Phillips An et al., 2013, Plos One [42]	ART	MSM	UK	5	6	143
Monteiro Jfg et al., 2015, Int J Public Health [43]	Prevention Packages	PWID; NU; FSW	Cabo Verde	7	3	6
Phillips An et al., 2015, Aids [44]	Single Intervention	MSM	UK	4	2	43
Escudero Dj et al., 2016, Aids [17]	Transmission Dynamics	PWID	New York City, NY—US	7	2	8
Goodreau Sm et al., 2017, Lancet Hiv [45]	Transmission Dynamics	MSM; Racial Disparities	Atlanta, GA—US	4	6	64
Gantenberg Jr et al., 2018, Plos One [46]	PrEP	MSM	Rhode Island—US	11	5	12
Marshall Bdl et al., 2018, Lancet Hiv [47]	PrEP	MSM	Atlanta, GA—US	4	3	25
Jenness Sm et al., 2020, Aids [48]	Prevention Packages	MSM	Atlanta, GA, US	4	4	18

LcoCi: Local Co-Citations. LCs: Local Citations, computed as the number of citations from papers collected for the review. GCs: Global Citations, the overall paper citations till the date of extraction.

PrEP: pre-exposure prophylaxis. MSM: Men who have sex with men. PWID: People who inject drugs. WSW: women who have sex with women. NU: non-drug users. NIDU: non-injection-drug.

Beyond the Global study of Beyrer et al. [39], three studies analyzed countries: two studies about UK, one about Uganda and other about Cabo Verde. Other six studies deal with lower urban dimensions of the United States. They evaluate ART [42], PrEP [46, 47], Prevention Packages [40, 43, 48], Transmission Dynamics [17, 39, 45], and *S*ingle Interventions [41, 44]. In the following sections we will explore the main papers under the light of the overall categories’ description.

#### Aims

We classified 14 aim categories. Some articles have more than one category what let us with a total of 171 categories appearances. Transmission Dynamics (42/171), PrEP (20/171), Single Intervention (20/171), Cost-Effectiveness (19/171), and Prevention Packages (18/171) are the five most frequent categories. We described the complete information about the aims in Table C in S3 Appendix. Fig 2 identifies their appearances by publication’s year.

We can verify the Transmission Dynamics, beyond to be the most frequent goal in the analysis, was an interest of earlier papers (till 2018). Single Interventions type of articles follow similar pattern. This make sense because the transmission dynamic is the ABM core for HIV or other infectious diseases. Cost-Effectiveness, and PrEP concentrates after 2016. This kind of study can be an update, or the next step in the modelling challenge. The main papers are focused on three types of these articles in a similar way. Transmission Dynamics and Prevention Packages are the goal of the earlier articles. Cost-Effectiveness, PrEP and other interventions of the later.

In addition, the ABM applications for epidemiologic issues are usually a network model or structure of interactions [26]. All models we investigated in this review fit on this assumption. The interaction between agents is represented in a network fashion with some particularities. This helps to represent the transmission dynamic features. For example, Beyrer et al. [39] compare different scenarios based on the type of coital act (vaginal, role segregation, UAI). This helped them to identify the relation of HIV burden on MSM and UAI. Goodreau et al. [45] focus on the burden on Black African-Americans and identified the insufficiency of race assortative to explain the HIV burden on them–possible explanations are the care cascade gap and co-occurrence of other Sexual Transmission Infections (STIs). They could also design a very specific feature on the transmission dynamic path, such as Acute HIV Infection (AHI) in PWID [17]. Or a single intervention related to the transmission dynamics: *i*.*e*. reduction on concurrence partnership in Uganda [41]; the association between ART coverage and risk behaviors [42]; and the increasing in testing rates in the UK [44].

Other main papers evaluate PrEP strategies. Marshall et al. [47] simulate long-acting injectable PrEP and identified they could avoid 2,374 new infections with 35% coverage. Gantenberg et al. [46] explore different criteria to implement PrEP, targeting a 25% reduction on Rhode Island. Achieved only by scenarios with more than 15% of coverage (the real coverage is around 5% [46]). Goedel et al. [49] explore PrEP interventions scenarios based on a metric design to achieve a dual goal of reduce incidence and disparity between White and Black MSM.

As the care cascade strategies made clear, there is no silver bullet against HIV. Even more, combined interventions can reproduce adverse effects than the ones produced on individual randomized-control trials (RCT) [40, 50]. Marshall et al. identified the combined interventions could be more effective on IDU of NYC [40], while Monteiro et al. [43] find similar results in Cabo Verde. Jenness et al. [48], studying the Ending HIV Epidemic (EHE) strategy in Atlanta (US), suggest the EHE should drastically improve their care cascade and focus on black MSM.

#### Populations

**Fig 3:**
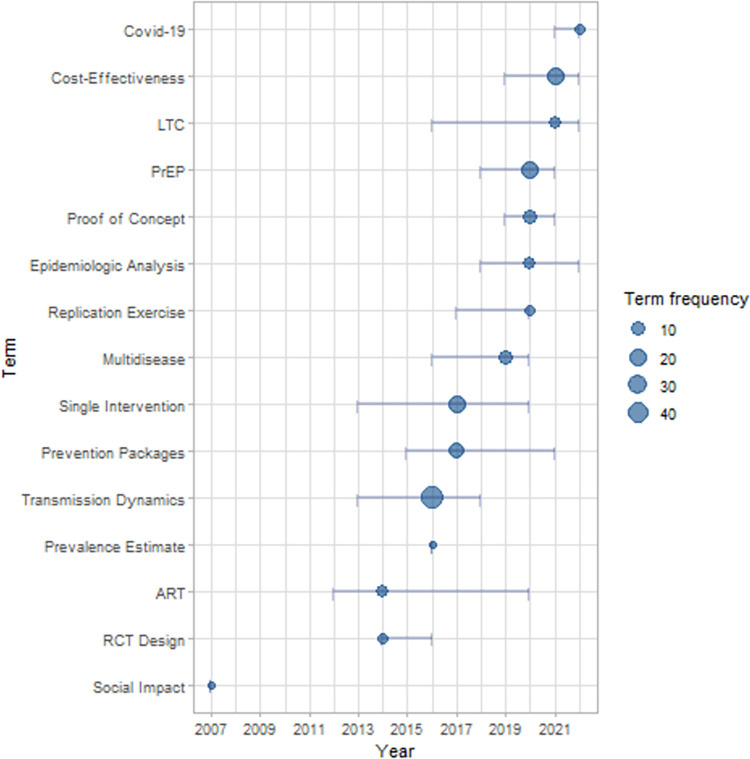
Distribution by year of population appearances in the studies. The dots represent the median publication year of the term appearance. Their size represents the term frequency. The lines are the quantiles 25 till 75 of year of appearance. PLWH: People Living with HIV. MSM: Men who have sex with men. FSW: Female sex workers. PWID: People who inject drugs. WSW: women who have sex with women. NU: non-drug users. NIDU: non-injection-drug.

We identified 18 studied populations on the 154 articles (see Fig 3). Most studies model men who have sex with men (MSM) (41/154), and heterosexual (27/154) populations. When we collected the overall appearances of populations (183), the five most frequent are: MSM (55/183), Heterosexual (35/183), Representative (20/183), PWID (18/183), PLWH (12/183), and Racial Disparities (12/183).

**Fig 4:**
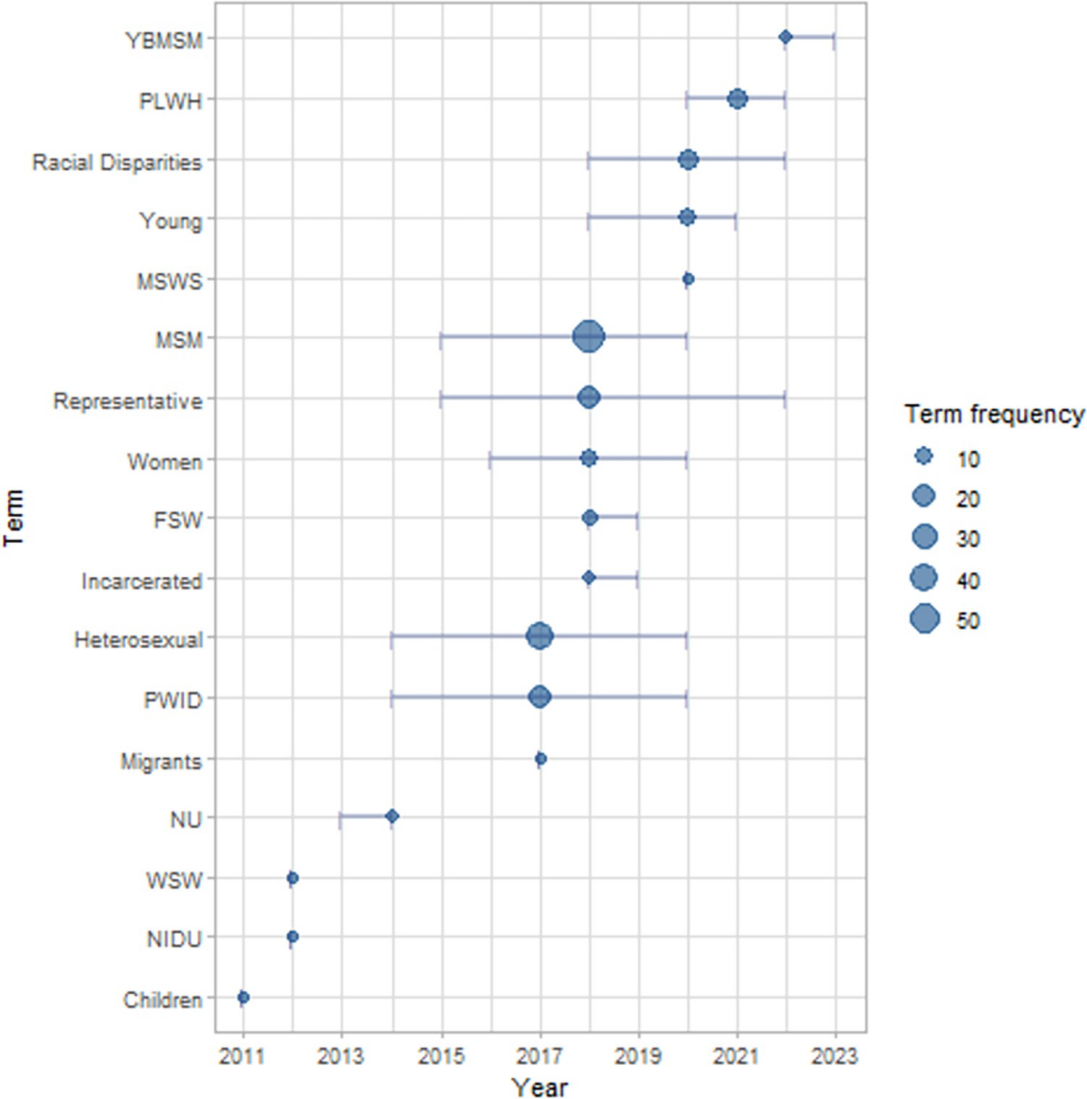
Distribution by year of aims appearances in the studies. The dots represent the median publication year of the term appearance. Their size represents the term frequency. The lines are the quantiles 25 till 75 of year’s appearance. LTC: Linkage to Care. PrEP: pre-exposure prophylaxis. ART: Antiretroviral Therapy. RCT Design: Random Control Trials.

We aggregated the studies focused on black subpopulations in the Racial Disparity category to highlight this thematic. Other populations categories appeared just few times, *e*.*g*., Women, Cisgender Male Sex Worker (MSWS), Women who have sex with Women (WSW), non-drug users and non-inject drug users (NU, and NIDU). In Fig 4, we show their distribution over years. We noticed some populations appeared more in studies published after 2019: (MSWS), pregnant women, female sex workers (FSW), people living with HIV (PLWH). While MSM, heterosexual, young, PWID, and representative populations are constant in the literature. The focus on Racial Disparities and PLWH are new trends in the subpopulation category. A detailed information can be found on Tables D and E in S3 Appendix.

Furthermore, the MSM population leads to two formalizations: sexual behavior and biologic risk. By biologic risk we mean the increased risk of UAI and the relative risk regarding to the sexual role (insertive, receptive and versatile). Beyrer et al. [39] discuss these features. They are part of the standard characterization of MSM population on ABM applications. Some interesting characterizations can also be mentioned about the condom use (diminishing by age and time of the relation [39]), partner selection (main, regular and causal [51]), and the assortative mixing by age [46] and by race[45].

Other main studies deal with interactive populations, including male and female heterosexuals, MSM, woman who have sex with woman (WMW), and drug users (DU) to represent the NYC epidemic [17, 40]. The DU status is characterized affecting the network formation and the probability of infection. In this case, the work of Escudero [17] is an extension Marshall [47] model focusing on PWID.

#### Regions

We identified 49 different locations. Most of them, discussed the countries of United States (15/154), and South Africa (15/154). To improve this understanding, we identified the geographic dimension of each locality studied. 62/154 studies applied an ABM to explore the HIV epidemics on countries, 40/154 studies modelled HIV epidemic in Cities (Table 3).

**Table 3:** Geographic dimensions studied.

Geographic Dimension	Articles
Country	62
City	40
State	19
Macro-region	13
Micro-Region	4
Region	3
Continent	1
Global	1
Neighborhood	1

Most of studies that focused on countries are from other countries than United States. While studies in United States are focused on lower dimensions, mostly urban regions. Six studies in the main papers are focused on specific dynamics that could be unnoticed in a country scale. Other possible explanation is related to the data available for this analysis: local detailed data is restricted.

In Table 4, we identified the countries studied independently of the geographic dimension (147 appearances). United States (57/147) counts for more than a third of the countries studied. Followed by South Africa (27/147), Kenya (11/147), and Australia (7/147).

**Table 4:** 10 most frequent countries under study.

Country	Frequency
US	57
South Africa	27
Kenya	11
Australia	7
Netherlands	7
Uganda	6
UK	6
Zambia	6
Zimbabwe	3
Benin	2

Frequency of the 10 countries most studied in the dataset. Studies that explored larger dimensions where exclude of these counting. When one study address more than one country, both where computed.

The studies concentration on the United States and South Africa can be explained by the burden of the epidemic on these countries and the availability of data. But, also, by the concentration of researchers’ networks on these two countries, a trend seen in other mathematical models applications as well [52].

#### Overview design and details protocol and limitations

The development of ABM demands interdisciplinary work. Therefore, some communication difficulties emerge. Network analysis, coding capability, biomedical, epidemiological, sociological, and statistical knowledge are some examples of the kind of knowledge applied on them. These different disciplines communicate in different ways. The literature about ABM already has identified this challenge [26, 25, 38, 37] and some efforts were made on the standardization of the results’ communication with ABM [37, 38]. The Overview Design and Details (ODD) protocol was developed to standardize the description of individual-based models [37, 38]. We verified that few studies applied the ODD protocol (6/154) and a quarter of the articles (34/154) did not mention the study limitations explicitly.

In particular, between the main articles, just Marshall et al. [40], mentioned the ODD protocol. Nevertheless, the studies of Escudero et al. [17], and Marshall et al. [47] have a similar structure, without mentioning the protocol. Gantenberg et al. [46] mentioned the protocol in the supplementary information, scaping from our search strategy (that focused only on mentions directly into the paper). The study of Beyrer et al. [39] differs in structure with a long description of the comprehensive review they made about the risk factors associated with the HIV.

When we looked to the limitations, just Goodreau et al. [45] didn’t mentioned it. Some limitation mentioned are case-specific. Like the possibility to use different kinds of PrEP by agent [47], or the behavioral risk compensation (e.g. the decreasing of condom use when initiate PrEP) [46]. Others are common issues in the literature: limited data, system size effect and geographical mobility. However, the main studies have specific data to explore their questions, they still must fill gaps with external data or assumptions. Also, computational power still limits the possibility to explore larger number of agents (system size effect) or implement more complex characterizations (*e*.*g*. geographical mobility).

## Discussion

The results of our review on the use of ABM to model HIV/AIDS epidemic shows an important heterogeneity of applications. This demonstrate the versatility of this kind of modeling to evaluate interventions to overcome the challenges of the current stage of HIV epidemic [40]. We identified a literature focused on the topics of Transmission Dynamics, PrEP, Cost-Effectiveness, and Prevention Packages. These studies are mainly about the United States and South Africa, and the most of them represent MSM, Heterosexual, PWID populations. We also notice a growing discussion related to Racial Disparities. These applications take advantage of an individual-based framework where the bottom-up design with heterogeneous interactions between agents produce aggregate outputs and emergent properties.

As we mentioned, most of the studies focused on Transmission Dynamics and Prevention Packages were published before 2018. We noticed a transition in the themes explored by the modelers due to the evolution of the model characteristics. The transmission dynamics is the core of the ABM for HIV and once done, it is an infrastructure capable to evolve to explore other themes. Many of the models are made with a modular structure allowing this updates, improving the previous models [53–55]. Furthermore, part of the LcoCi are, in fact, citations from other studies of the same group, or extensions of previous models. This reflects an internal bias in the literature, but also a characteristic to be discuss. We believe this is related to internal characteristics of the HIV ABM literature: concentrated in fewer groups. This literature still grows more on direct collaborations (co-authors) than in communication efforts (published articles).

The concentration of studies on the United States and South Africa could be explained by the evolving availability of resources: high computational power, model development, and empirical data for calibration and validation [24, 56] (avoiding bias from external sources [46, 49, 57, 58]). This can also explain why the geographic dimension of study tend to be smaller regions. One example are the studies focused on PrEP interventions. They are restricted to specific populations, more representative on lower geographic dimensions. In addition, lower dimensions and specific populations are alternatives to highlight more restrict dynamics.

No study explores Latin America (Beyrer et al. [39] mentioned Peru, but on a global perspective). However, most studies deals with US reality. In fact, some cities like Atlanta, or New York, have more studies than some sub-Saharan countries (*e*.*g*., Botswana, Kenya, Uganda, or Zambia). Under a historic perspective, we can identify a bifurcation in the studies from the national perspective on transmission dynamics, and prevention packages among MSM, and heterosexual populations. To a more specific, focused on urban dynamics, targeted intervention, and particular subpopulation. This demonstrates the range of ABM applications: from local dynamics till countrywide. Alternatively, the Prevention Packages kind of study is especially interesting to be explored by ABM [40, 51]. The capacity to analyze combinations of interventions is a great potentiality of ABM for policymaking purposes. Combined interventions of more accessible instruments could be a better choice under budget and resources restrictions.

In addition, the history of the HIV is marked by stigma towards the homosexual population [59], while the transmission dynamics lays down also on the social structures. Racism is one transversal dimension that expose these populations to risk behaviors [16] or limited access to test and treatment [15]. We decided to aggregate studies that explore different subpopulations related to Black/African-American populations (MSM, Heterosexual, Incarcerated, Woman, Young) as a population topic (Racial Disparities) to give emphasis on this racial approach. An emerging theme in the applications.

We highlight this point because, most of the models focused on MSM representing this population by its biological and behavior risks. Named: sex role, number of partners and practice of UAI. Racial Disparities approach explore a more sociological discussion inserting race assortative matching [45], how the massive incarceration of the Black/African American men could affect the incidence on Black/African American Women [59], or the PrEP coverage necessary to reduce race disparities [49]. This adds another layer on the model representation of the epidemic. Closer to the Rainbow model perspective [12, 60].

Unfortunately, no other social features related to SDH were explored. Just one study aimed the social impact of HIV [61]. However, this is different than explore how these social features impact the HIV/AIDS epidemic. Also, even with more than 30 studies published after 2021, just four of them explored the implications of Covid-19 on the HIV epidemics [55, 62–64]–e.g., the effects on test, treatment, and social conditions. The implications of climate change, and growing poverty were also missed in the debate. The Racial Disparities discussion is a good example of how ABM interface between public health and social structure can provide insights for policy designs to “make sure no one is left behind”[5].

All this complexity requires clear understanding of model features, results, and limitations. Nonetheless, a protocol for IBM description already exists. The ODD protocol [37, 38] was developed for ecological studies and was poorly adopted by the ABM studies identified here. We can think the ODD structure is not well suited for public health journals, neither diffused on the area yet. Although, we also found model’s limitations poorly declared. The way ABM are described still challenges researchers and limit their influence [65].

Also, several limitations are present in this study: the selection of the main papers in the literature, identified by a local co-citation analysis can neglect some recent studies [35]; the computation of LcoCi depends on the bibliometric data available; we considered ABM and IBM as synonyms to incorporate more studies [26, 66]; Our strategy to find ODD declaration can omit some cases as the Gantenberg et al. [46] case. At last, we avoid explore model details to provide a broader view of ABM applications on HIV epidemics. Future research can step forward to the modelling details, such as quality of the data used, calibration methods, validations process, sensitivity analysis, behavior characterization, risk assumptions, network formation, system size effects and other challenges [26].

In conclusion, ABM have demonstrated to be a useful methodology for the modelling of the HIV/AIDS pandemic as a complex phenomenon, by explicitly represent heterogenous interactions of heterogenous individuals on different environments. This methodology has the potential to integrate a wide range of social determinants of health into the modelling of HIV/AIDS. However, just a few of them have been included until now with the notable absence of socioeconomic vulnerabilities. The increasing availability of data (and big data), and the surge of high-performance computing worldwide could contribute to the development of large-scale ABM, or models able to include socioeconomic vulnerability aspects in the modeling framework.

## Supporting information

S1 Appendix Search strategy. (DOCX)

S2 Appendix Data charting and detailed categories. (DOCX)

S3 Appendix Complete tables. (DOCX)

S4 Appendix Checklist of PRISMA extension for scoping review. (PDF)

S5 Appendix Script files. The database and analysis performed for the study.
(ZIP)

S1 Table Studies dataset with charted variables. (DOCX)
