# Supplementary material for: Modelling HIV/AIDS epidemiological complexity: A scoping review of Agent-Based Models and their application
Source: PLoS One. 2024 Feb 2;19(2):e0297247. doi: 10.1371/journal.pone.0297247 (PMC10836677; doi:10.1371/journal.pone.0297247)
Supplement: S1 Appendix — (DOCX) [file pone.0297247.s001.docx]

**S1 Appendix -** **Search strategy**

Our search strategy was thought to restrict studies that focuses only on HIV diseases that applied agent-based models on epidemiological studies. For this reason, we specified the MESH term “HIV” or “human immunodeficiency virus” in the title, and “agent-based” or “agent based” in all sections of papers in the PubMed, Web of Science and Scopus databases.

We considered just ABM which is the most common term used to epidemiological models based on individuals and can be found in the Systems Analysis MESH term (<https://www.ncbi.nlm.nih.gov/mesh/68013597>).

We collected the data on August 1^st^, 2023, without date limit and restricting to articles results. When possible, we also remove the reviews. Bellow, we detailed the search syntax of each database, their results and the information extracted for the analysis and review.

***PubMed***

*Search*

(("hiv"[Title] OR "human immunodeficiency virus"[Title]) AND ("agent-based"[All Fields] OR "agent based"[All Fields] OR "individual-based"[All Fields] OR "individual based"[All Fields])) NOT (("hiv"[Title] OR "human immunodeficiency virus"[Title]) AND ("agent-based"[All Fields] OR "agent-based"[All Fields] OR "individual-based"[All Fields] OR "individual based"[All Fields]) AND "review"[Publication Type])

*Results* 197

*Extraction*

PubMed file.

***Web of Science***

*Search*

((TI=(HIV) OR TI=(“human immunodeficiency virus”))) AND (ALL=("agent-based") OR ALL=("agent based") OR ALL=("individual-based") OR ALL=("individual based"))

Filtered by Type of document: Article

*Results* 198

*Extraction*

Complete Bibtex file.

***Scopus***

*Search*

( TITLE ( "hiv" ) OR TITLE ( "human immunodeficiency virus" ) ) AND ( TITLE-ABS-KEY ( "AGENT-BASED" ) OR TITLE-ABS-KEY ( "AGENT BASED" ) OR TITLE-ABS-KEY ( "INDIVIDUAL-BASED" ) OR TITLE-ABS-KEY ( "INDIVIDUAL BASED" ) ) AND ( LIMIT-TO ( DOCTYPE , "ar" ) )

Filter: Document Type = “Article”

*Results* 257

*Extraction*

Complete Bibtex file.

***Manually searched***

One of the reviewers notes that some papers were missed in our search strategy. We add them manually by searching directly on the repositories of Web of Science, Scopus and PubMed. Unfortunately, two of them were available just on PubMed which means we don’t have citated references data that we need for the co-citation analysis:

***PubMed***

- Jenness SM, Goodreau SM, Rosenberg E, et al. Impact of the Centers for Disease Control's HIV Pre-Exposure Prophylaxis Guidelines for Men Who Have Sex with Men in the United States. /The Journal of Infectious Diseases/. Published online 2016-07:jiw223. doi:10.1093/infdis/jiw223

- Jenness SM, Le Guillou A, Chandra C, et al. Projected HIV and Bacterial STI Incidence Following COVID-Related Sexual Distancing and Clinical Service Interruption. /The Journal of Infectious Diseases/. 2021;223(6):1019-1028. doi:10.1093/infdis/jiab051

***Web of Science***

Kasaie P, Pennington J, Shah MS, et al. The Impact of Pre-Exposure Prophylaxis among Men Who Have Sex with Men: An Individual-Based Model. /Journal of Acquired Immune Deficiency Syndromes/. 2017-03;75(2):175-183. doi:10.1097/QAI.0000000000001354

Jenness SM, Sharma A, Goodreau SM, et al. Individual HIV Risk versus Population Impact of Risk Compensation after HIV Preexposure Prophylaxis Initiation among Men Who Have Sex with Men. /PLoS ONE/. 2017-01;12(1):e0169484. doi:10.1371/journal.pone.0169484

Jenness SM, Johnson JA, Hoover KW, Smith DK, Delaney KP. Modeling an Integrated HIV Prevention and Care Continuum to Achieve the Ending the HIV Epidemic Goals. /AIDS/. 2020-09-08;34(14):2103-2113. doi:10.1097/QAD.0000000000002681

**Merging databases**

We read the databases with Bibliometrix package in R software. After reading and converting the files to a “data.frame” type, we applied a “full_join”, merging the data bases checking for missing DOI codes and distinct the articles by them. We use the resulting table to proceed a manual filtering of the papers to our selection criteria.

**Missing DOI**

We found 25 articles with missing DOI and manually included nine of them.

We do not identify DOI code for TEN articles. For those, we identified the PMID code, from PubMed, or web address for two papers published on JASS. However, five articles remain without an identifier and we set them as NA:

[1] "COMPUTATIONAL MODEL OF HIV-1 ESCAPE FROM THE CYTOTOXIC T LYMPHOCYTE RESPONSE"

[2] "AGENT-BASED SIMULATION OF HIV PROPAGATION AMONG INTRAVENOUS DRUG USERS IN SOCIAL CIRCLES NETWORK"

[3] "USING GENETIC ALGORITHMS VERSUS LINE SEARCH OPTIMIZATION FOR HIV PREDICTIONS"

[4] "ANTI-HIV ACTIVITY OF PHOSPHOROTHIOATE OLIGODEOXYNUCLEOTIDES CONTAINING CONSECUTIVE G SEQUENCES"

[5] "HUMAN IMMUNODEFICIENCY VIRUS (HIV) AND ACQUIRED IMMUNODEFICIENCY SYNDROME (AIDS) CASE REPORTING IN THE WORLD HEALTH ORGANIZATION EUROPEAN REGION IN 2006."

[6] "HUMAN IMMUNODEFICIENCY VIRUS (HIV) AND ACQUIRED IMMUNODEFICIENCY SYNDROME (AIDS) CASE REPORTING IN THE WORLD HEALTH ORGANIZATION EUROPEAN REGION IN 2006."

**Deduplicating**

We suppressed the duplicates taken Web of Science and Scopus database as reference - because they provide references and citations - resulting in 15 articles that came just from PubMed:

- 197 from Web of Science
- 71 from Scopus
- 15 from PubMed

**Manual Checking for Inclusion Criteria**

As inclusion criteria we specified that the articles should have an application of an Agent-based Model and be focused on epidemiological outputs.

1) studies that focused on epidemiological outputs of HIV/AIDS

2) studies that provide an application of Agent-Based Model, or an Individual-Based Model

*Double Checking*

We made a double-checking procedure with two researchers where 23 divergences arise. After a new double-checking we end with 11 divergences. We discuss each of them individually and agreed with a final number. Part of the divergences are due to the lack of specification on the first criteria that could be interpretative from different perspectives.

The manual checking resulted in 154 papers:

- 139 from Web of Science
- 7 from PubMed
- 8 from Scopus

The 129 rejected papers:

- 49 were rejected by criteria 1 and 2;
- 33 due the criteria 1
- 41 due the criteria 2
- 6 weren’t aN article or Other reasons

**Co-citation Analysis**

To perform a co-citation analysis we use the Bibliometrix package on R software (<https://www.bibliometrix.org/vignettes/Introduction_to_bibliometrix.html>) with the Bibtex file resulted from the full join of the sources mentioned above.

The co-citation results can be found as the papers with more than one local citations (LCS) in the Table A.

**Table A. Co-Citations, Local Citations, and Global Citations**

| Study | Year | DOI | LcoCi | LCs | GCs |
| --- | --- | --- | --- | --- | --- |
| NIYUKURI D, 2021, MATHEMATICS | 2021 | 10.3390/math9212645 | 0 | 0 | 2 |
| GRAW F, 2012, EPIDEMICS | 2012 | 10.1016/j.epidem.2012.04.002 | 0 | 0 | 29 |
| BUCHANAN ALL, 2022, EPIDEMIOL INFECT | 2022 | 10.1017/s0950268822001650 | 0 | 0 | 0 |
| SINGH S, 2021, MATH BIOSCI ENG | 2021 | 10.3934/mbe.2021109 | 0 | 0 | 3 |
| VERMEER W, 2020, JASSS | 2020 | 10.18564/jasss.4352 | 0 | 0 | 4 |
| HUANG CY, 2015, COMPUT MATH METHOD MED | 2015 | 10.1155/2015/867264 | 0 | 0 | 4 |
| DEMEULEMEESTER R, 2022, BMC HEALTH SERV RES | 2022 | 10.1186/s12913-022-07859-w | 0 | 0 | 2 |
| VERMEER W, 2022, PLOS ONE | 2022 | 10.1371/journal.pone.0274288 | 0 | 0 | 0 |
| RENIERS G, 2015, DEMOGR RES | 2015 | 10.4054/demres.2015.33.15 | 0 | 0 | 7 |
| LUO W, 2018, JMIR PUBLIC HEALTH SURVEILL | 2018 | 10.2196/publichealth.9357 | 0 | 0 | 10 |
| LABS J, 2022, OPEN FORUM INFECT DIS | 2022 | 10.1093/ofid/ofac274 | 0 | 0 | 1 |
| GOPALAPPA C, 2017, MED DECIS MAK | 2017 | 10.1177/0272989x16668509 | 0 | 0 | 42 |
| MITTLER JE, 2019, PLOS COMPUT BIOL | 2019 | 10.1371/journal.pcbi.1007561 | 0 | 0 | 4 |
| CHAN PA, 2019, AIDS PATIENT CARE STDS | 2019 | 10.1089/apc.2019.0064 | 0 | 0 | 14 |
| MCKAY VR, 2021, AIDS BEHAV | 2021 | 10.1007/s10461-020-03051-5 | 0 | 0 | 1 |
| SINGLETON AL, 2020, AIDS PATIENT CARE STDS | 2020 | 10.1089/apc.2020.0151 | 0 | 0 | 4 |
| ALAM SJ, 2007, JASSS | 2007 | https://www.jasss.org/10/4/7.html | 0 | 0 | 16 |
| JACKA BP, 2022, JAIDS | 2022 | 10.1097/qai.0000000000003093 | 0 | 0 | 0 |
| WHITE PJ, 2014, J INFECT DIS | 2014 | 10.1093/infdis/jiu470 | 0 | 0 | 9 |
| NICHOLS BE, 2022, J INT AIDS SOC | 2022 | 10.1002/jia2.26020 | 0 | 0 | 1 |
| RICHARDSON L, 2012, SOC SCI RES | 2012 | 10.1016/j.ssresearch.2011.12.004 | 0 | 0 | 11 |
| ZANG X, 2022, AIDS | 2022 | 10.1097/qad.0000000000003199 | 0 | 0 | 0 |
| ABUELEZAM NN, 2016, AM J EPIDEMIOL | 2016 | 10.1093/aje/kwv344 | 0 | 0 | 12 |
| GOEDEL WC, 2018, JAIDS | 2018 | 10.1097/qai.0000000000001817 | 0 | 0 | 40 |
| DES JARLAIS D, 2022, DRUG ALCOHOL DEPEND | 2022 | 10.1016/j.drugalcdep.2022.109573 | 0 | 0 | 2 |
| HENDRICKX DM, 2021, EPIDEMICS | 2021 | 10.1016/j.epidem.2021.100474 | 0 | 0 | 0 |
| SCOTT N, 2018, J INT AIDS SOC | 2018 | 10.1002/jia2.25059 | 0 | 0 | 15 |
| ROBERTS ST, 2016, J INT AIDS SOC | 2016 | 10.7448/ias.19.1.20864 | 0 | 0 | 5 |
| KHATAMI SN, 2021, MATH BIOSCI ENG | 2021 | 10.3934/mbe.2021380 | 0 | 0 | 1 |
| LEE F, 2023, MED CARE | 2023 | 10.1097/mlr.0000000000001772 | 0 | 0 | 0 |
| MILWID RM, 2022, BMC INFECT DIS | 2022 | 10.1186/s12879-022-07207-7 | 0 | 0 | 3 |
| BOREN D, 2014, STAT MED | 2014 | 10.1002/sim.6193 | 0 | 0 | 6 |
| ISAAC AG, 2019, J ECON INTERACT COORD | 2019 | 10.1007/s11403-018-00234-1 | 0 | 0 | 0 |
| KATZ DA, 2021, SEX TRANSM DIS | 2021 | 10.1097/olq.0000000000001485 | 0 | 0 | 0 |
| NEAIGUS A, 1999, PUBLIC HEALTH | 1999 | 9722819 | 0 | 0 | 2 |
| JACOBSON EU, 2023, AIDS CARE-PSYCHOL SOCIO-MED ASP AIDS-HIV | 2023 | 10.1080/09540121.2022.2147478 | 0 | 0 | 0 |
| OSETINSKY B, 2019, HEALTH AFF | 2019 | 10.1377/hlthaff.2018.05287 | 0 | 0 | 10 |
| DOMBROWSKI K, 2017, AIDS BEHAV | 2017 | 10.1007/s10461-016-1568-6 | 0 | 0 | 11 |
| GOEDEL WC, 2020, SCI REP | 2020 | 10.1038/s41598-020-62694-5 | 0 | 0 | 6 |
| TULLY S, 2015, SCI REP | 2015 | 10.1038/srep15411 | 0 | 0 | 14 |
| FREEMAN EE, 2009, VACCINE | 2009 | 10.1016/j.vaccine.2008.11.074 | 0 | 0 | 40 |
| WILSON DP, 2011, SEX TRANSM INFECT | 2011 | 10.1136/sextrans-2011-050002 | 0 | 0 | 8 |
| SINGLETON AL, 2021, EPIDEMICS | 2021 | 10.1016/j.epidem.2020.100426 | 0 | 0 | 2 |
| PEEBLES K, 2021, SCI REP | 2021 | 10.1038/s41598-021-85487-w | 0 | 0 | 1 |
| JOHNSON LF, 2018, EPIDEMIOL INFECT | 2018 | 10.1017/s0950268818000961 | 0 | 0 | 5 |
| JOHNSON LF, 2020, PLOS ONE | 2020 | 10.1371/journal.pone.0242595 | 0 | 0 | 2 |
| KASAIE P, 2022, ANN EPIDEMIOL | 2022 | 10.1016/j.annepidem.2021.08.021 | 0 | 0 | 2 |
| GOYAL R, 2021, JAIDS | 2021 | 10.1097/qai.0000000000002546 | 0 | 0 | 3 |
| ALTHOFF KN, 2022, AIDS | 2022 | 10.1097/qad.0000000000003128 | 0 | 0 | 9 |
| HAMILTON DT, 2022, JAIDS | 2022 | 10.1097/qai.0000000000003013 | 0 | 0 | 1 |
| BECK EC, 2015, JAIDS | 2015 | 10.1097/qai.0000000000000733 | 0 | 0 | 44 |
| SCHNEIDER K, 2011, AIDS | 2011 | 10.1097/qad.0b013e3283466fab | 0 | 0 | 19 |
| LEVASSEUR MT, 2018, JAIDS | 2018 | 10.1097/qai.0000000000001555 | 0 | 0 | 27 |
| BERSHTEYN A, 2018, INFECT DIS MODEL | 2018 | 10.1016/j.idm.2018.04.001 | 0 | 0 | 11 |
| ROMERO-SEVERSON EO, 2013, EPIDEMIOLOGY | 2013 | 10.1097/ede.0b013e318294802e | 0 | 0 | 12 |
| REITSEMA M, 2020, AIDS | 2020 | 10.1097/qad.0000000000002469 | 0 | 0 | 9 |
| KOENIG LJ, 2023, AIDS | 2023 | 10.1097/qad.0000000000003536 | 0 | 0 | 1 |
| WANG R, 2014, CLIN TRIALS | 2014 | 10.1177/1740774514523351 | 0 | 0 | 19 |
| LEE F, 2022, INT J DRUG POLICY | 2022 | 10.1016/j.drugpo.2022.103628 | 0 | 0 | 1 |
| GOYAL R, 2021, JAIDS-a | 2021 | 10.1097/qai.0000000000002547 | 0 | 0 | 5 |
| JEWELL BL, 2021, JAIDS | 2021 | 10.1097/qai.0000000000002684 | 0 | 0 | 2 |
| ANDERSSON E, 2019, EUROSURVEILLANCE | 2019 | 10.2807/1560-7917.es.2019.24.14.1800203 | 0 | 0 | 2 |
| HAMILTON DT, 2023, EPIDEMICS | 2023 | 10.1016/j.epidem.2023.100696 | 0 | 0 | 0 |
| MILALI MP, 2023, FRONT REPROD HEALTH | 2023 | 10.3389/frph.2023.1144217 | 0 | 0 | 0 |
| WOOD D, 2018, JAIDS | 2018 | 10.1097/qai.0000000000001603 | 0 | 0 | 3 |
| SMITH JA, 2017, CONTRACEPTION | 2017 | 10.1016/j.contraception.2016.12.003 | 0 | 0 | 9 |
| KASAIE P, 2019, BMJ OPEN | 2019 | 10.1136/bmjopen-2018-023453 | 0 | 0 | 5 |
| BASTEN M, 2018, AIDS | 2018 | 10.1097/qad.0000000000001803 | 0 | 0 | 22 |
| ABBAS UL, 2019, PLOS ONE | 2019 | 10.1371/journal.pone.0218649 | 0 | 0 | 0 |
| HAMILTON DT, 2023, BMC PUBLIC HEALTH | 2023 | 10.1186/s12889-023-15563-5 | 0 | 0 | 0 |
| GOUNTAS I, 2019, J VIRAL HEPATITIS | 2019 | 10.1111/jvh.13178 | 0 | 0 | 7 |
| OMORI R, 2017, AIDS | 2017 | 10.1097/qad.0000000000001542 | 0 | 0 | 21 |
| TWEYA H, 2016, AIDS | 2016 | 10.1097/qad.0000000000001009 | 0 | 0 | 12 |
| MCCREESH N, 2018, PLOS ONE | 2018 | 10.1371/journal.pone.0196480 | 0 | 0 | 1 |
| SHARMA M, 2021, LANCET HIV | 2021 | 10.1016/s2352-3018(20)30279-4 | 0 | 0 | 9 |
| ROBERTS DA, 2022, J INT AIDS SOC | 2022 | 10.1002/jia2.26034 | 0 | 0 | 1 |
| CAMBIANO V, 2018, LANCET INFECT DIS | 2018 | 10.1016/s1473-3099(17)30540-6 | 0 | 0 | 64 |
| KIM HY, 2020, J INT AIDS SOC | 2020 | 10.1002/jia2.25432 | 0 | 0 | 4 |
| CHEMAITELLY H, 2022, LANCET HIV | 2022 | 10.1016/s2352-3018(22)00100-x | 0 | 0 | 2 |
| THOMAS R, 2021, LANCET GLOB HEALTH | 2021 | 10.1016/s2214-109x(21)00034-6 | 0 | 0 | 13 |
| PHILLIPS AN, 2021, LANCET GLOB HEALTH | 2021 | 10.1016/s2214-109x(21)00025-5 | 0 | 0 | 21 |
| KASAIE P, 2017, JAIDS | 2017 | 10.1097/qai.0000000000001354 | 0 | 0 | 34 |
| PROBERT WJM, 2022, Lancet HIV Health Educ. Behav. | 2022 | 10.1016/s2352-3018(22)00259-4 10.1177/1090198113487199 | 0 | 0 | 2 |
| VELEZ JD, 2015, RECOLETOS Multidiscip. Res. J. | 2015 | 10.32871/rmrj1503.02.10 | 0 | 0 | 0 |
| XIAO X, 2020, J. Assoc. NURSES AIDS CARE | 2020 | 10.1097/jnc.0000000000000122 | 0 | 0 | 4 |
| ZHANG J, 2022, Int. J. Biomath. | 2022 | 10.1142/s1793524522500292 | 0 | 0 | 0 |
| WOHL DA, 2013, Antimicrob. AGENTS Chemother. | 2013 | 10.1128/aac.01826-12 | 0 | 0 | 13 |
| RENTZ MF, 2017, Curr. HIV Res. | 2017 | 10.2174/1570162x15666171017121301 | 0 | 0 | 1 |
| NG KY, 2013, BMC Infect. Dis. | 2013 | 10.1186/1471-2334-13-90 | 0 | 0 | 20 |
| RAGONNET R, 2017, OPEN Forum Infect. Dis. | 2017 | 10.1093/oid/ofw235 | 0 | 0 | 3 |
| JOHNSON LF, 2023, BMC INFECT DIS | 2023 | 10.1186/s12879-023-08470-y | 0 | 0 | NA |
| PICKLES M, 2023, LANCET GLOB HEALTH | 2023 | s2214-109x(23)00206-1 | 0 | 0 | NA |
| PROBERT WJM, 2022, LANCET HIV | 2022 | s2352-3018(22)00259-4 | 0 | 0 | NA |
| VERMEER W, 2020, J ARTIF SOC SOC SIMUL | 2020 | 7 | 0 | 0 | NA |
| VANDEWALLE B, 2016, PLOS ONE | 2016 | 10.1371/journal.pone.0149007 | 0 | 0 | NA |
| phillJENNESS SM, 2016, J INFECT DIS | 2016 | doi.org/10.1093/infdis/jiw223 | 0 | 0 | NA |
| CUADROS DF, 2014, COMPUT BIOL MED | 2014 | 10.1016/j.compbiomed.2014.03.008 | 0 | 1 | 10 |
| TULLY S, 2013, J THEOR BIOL | 2013 | 10.1016/j.jtbi.2013.08.014 | 0 | 1 | 10 |
| MONTEIRO JFG, 2016, EPIDEMIOL INFECT | 2016 | 10.1017/s0950268815003180 | 5 | 1 | 8 |
| BUCHANAN AL, 2021, AM J EPIDEMIOL | 2021 | 10.1093/aje/kwaa239 | 2 | 1 | 6 |
| KASAIE P, 2018, SEX TRANSM DIS | 2018 | 10.1097/olq.0000000000000882 | 1 | 1 | 6 |
| ADAMS JW, 2018, BMC PUBLIC HEALTH | 2018 | 10.1186/s12889-018-6304-x | 2 | 1 | 17 |
| ADAMS JW, 2019, PLOS ONE | 2019 | 10.1371/journal.pone.0219361 | 3 | 1 | 4 |
| SAWERS L, 2017, AJAR-AFR J AIDS RES | 2017 | 10.2989/16085906.2017.1336105 | 2 | 1 | 5 |
| SELINGER C, 2019, VACCINE | 2019 | 10.1016/j.vaccine.2019.02.073 | 1 | 1 | 10 |
| HOARE A, 2012, SEX HEALTH | 2012 | 10.1071/sh10145 | 1 | 1 | 11 |
| SCHNEIDER K, 2014, CLIN INFECT DIS | 2014 | 10.1093/cid/cit946 | 2 | 1 | 45 |
| REITSEMA M, 2019, AIDS | 2019 | 10.1097/qad.0000000000002199 | 1 | 1 | 4 |
| HERBECK JT, 2016, VIRUS EVOL | 2016 | 10.1093/ve/vew028 | 2 | 1 | 15 |
| BOBASHEV G, 2019, PLOS ONE | 2019 | 10.1371/journal.pone.0215042 | 0 | 1 | 3 |
| REITSEMA M, 2020, SEX TRANSM INFECT | 2020 | 10.1136/sextrans-2018-053943 | 1 | 1 | 4 |
| BABIGUMIRA JB, 2022, BMJ OPEN | 2022 | 10.1136/bmjopen-2021-058636 | 1 | 1 | 1 |
| DIMITROV D, 2015, PLOS ONE | 2015 | 10.1371/journal.pone.0115528 | 1 | 1 | 6 |
| ESCUDERO DJ, 2017, BMC PUBLIC HEALTH | 2017 | 10.1186/s12889-017-4528-9 | 5 | 1 | 17 |
| SHARMA M, 2018, AIDS | 2018 | 10.1097/qad.0000000000001697 | 0 | 1 | 34 |
| NAKAGAWA F, 2017, AIDS | 2017 | 10.1097/qad.0000000000001329 | 1 | 1 | 2 |
| MCCREESH N, 2017, BMC INFECT DIS | 2017 | 10.1186/s12879-017-2420-y | 1 | 1 | 20 |
| DE VOS AS, 2013, ADDICTION | 2013 | 10.1111/add.12125 | 0 | 1 | 52 |
| MCCORMICK AW, 2014, PLOS ONE | 2014 | 10.1371/journal.pone.0098272 | 0 | 1 | 15 |
| WHITE RG, 2008, AIDS | 2008 | 10.1097/qad.0b013e32830e0137 | 2 | 1 | 70 |
| OLNEY JJ, 2016, LANCET HIV | 2016 | 10.1016/s2352-3018(16)30120-5 | 1 | 1 | 23 |
| PHILLIPS AN, 2019, J INT AIDS SOC | 2019 | 10.1002/jia2.25325 | 0 | 1 | 16 |
| JENNESS SM, 2021, J INFECT DIS | 2021 | 10.1093/infdis/jiab051 | 0 | 1 | NA |
| BROOKMEYER R, 2014, PLOS ONE | 2014 | 10.1371/journal.pone.0112668 | 2 | 2 | 35 |
| NAKAGAWA F, 2016, EPIDEMIOLOGY | 2016 | 10.1097/ede.0000000000000423 | 3 | 2 | 10 |
| GRAY RT, 2011, VACCINE | 2011 | 10.1016/j.vaccine.2011.06.061 | 1 | 2 | 10 |
| HERBECK JT, 2014, PLOS COMPUT BIOL | 2014 | 10.1371/journal.pcbi.1003673 | 2 | 2 | 22 |
| ESCUDERO DJ, 2016, AIDS | 2016 | 10.1097/qad.0000000000001218 | 7 | 2 | 8 |
| KHANNA AS, 2021, MATH BIOSCI ENG | 2021 | 10.3934/mbe.2021196 | 0 | 2 | 5 |
| KLEIN DJ, 2015, INT HEALTH | 2015 | 10.1093/inthealth/ihv011 | 2 | 2 | 11 |
| PHILLIPS AN, 2015, AIDS | 2015 | 10.1097/qad.0000000000000767 | 4 | 2 | 43 |
| GRAY RT, 2013, PLOS ONE | 2013 | 10.1371/journal.pone.0055449 | 2 | 2 | 44 |
| LECLERC PM, 2009, PLOS ONE | 2009 | 10.1371/journal.pone.0005439 | 1 | 2 | 31 |
| FREEMAN EE, 2007, SEX TRANSM INFECT | 2007 | 10.1136/sti.2006.023549 | 3 | 2 | 79 |
| GOPALAPPA C, 2017, AIDS | 2017 | 10.1097/qad.0000000000001653 | 1 | 3 | 8 |
| GOEDEL WC, 2020, CLIN INFECT DIS | 2020 | 10.1093/cid/ciz321 | 2 | 3 | 18 |
| GOEDEL WC, 2020, AIDS | 2020 | 10.1097/qad.0000000000002577 | 3 | 3 | 14 |
| MONTEIRO JFG, 2015, INT J PUBLIC HEALTH | 2015 | 10.1007/s00038-015-0676-9 | 7 | 3 | 6 |
| JANSSON J, 2012, PLOS ONE | 2012 | 10.1371/journal.pone.0038334 | 2 | 3 | 30 |
| BINGHAM A, 2021, SEX TRANSM DIS | 2021 | 10.1097/olq.0000000000001366 | 2 | 3 | 18 |
| KIM JH, 2010, EPIDEMIOLOGY | 2010 | 10.1097/ede.0b013e3181e6639f | 3 | 3 | 22 |
| MARSHALL BDL, 2018, LANCET HIV | 2018 | 10.1016/s2352-3018(18)30097-3 | 4 | 3 | 25 |
| SMIT M, 2015, LANCET INFECT DIS | 2015 | 10.1016/s1473-3099(15)00056-0 | 3 | 3 | 527 |
| JENNESS SM, 2017, PLOS ONE | 2017 | 10.1371/journal.pone.0169484 | 3 | 3 | 32 |
| BERSHTEYN A, 2016, INT HEALTH | 2016 | 10.1093/inthealth/ihw010 | 2 | 4 | 16 |
| MCCREESH N, 2012, SEX TRANSM DIS | 2012 | 10.1097/olq.0b013e318254c84a | 4 | 4 | 23 |
| SMITH JA, 2015, LANCET HIV | 2015 | 10.1016/s2352-3018(15)00016-8 | 2 | 4 | 53 |
| ORROTH KK, 2007, SEX TRANSM INFECT | 2007 | 10.1136/sti.2006.023531 | 3 | 4 | 56 |
| JENNESS SM, 2020, AIDS | 2020 | 10.1097/qad.0000000000002681 | 4 | 4 | 18 |
| GANTENBERG JR, 2018, PLOS ONE | 2018 | 10.1371/journal.pone.0199915 | 11 | 5 | 12 |
| GOODREAU SM, 2017, LANCET HIV | 2017 | 10.1016/s2352-3018(17)30067-x | 4 | 6 | 64 |
| PHILLIPS AN, 2013, PLOS ONE | 2013 | 10.1371/journal.pone.0055312 | 5 | 6 | 143 |
| MARSHALL BDL, 2012, PLOS ONE | 2012 | 10.1371/journal.pone.0044833 | 11 | 7 | 34 |
| BEYRER C, 2012, LANCET | 2012 | 10.1016/s0140-6736(12)60821-6 | 4 | 10 | 1056 |

LcoCi: Co-citation. LCs: Local Citations, computed as the number of citations from papers collected for the review. GCs: Global Citations, the overall paper citations till the date of extraction. NA: Missing information.

The following articles from PubMed doesn’t have detailed information about references and citations, and couldn’t be part of the co-citation analysis applied:

[1] "AN AGENT-BASED MODEL OF BINGE DRINKING, INEQUITABLE GENDER NORMS AND THEIR CONTRIBUTION TO HIV TRANSMISSION, WITH APPLICATION TO SOUTH AFRICA."

[2] "STRENGTHENING THE HIV PREVENTION CASCADE TO MAXIMISE EPIDEMIOLOGICAL IMPACT IN EASTERN ZIMBABWE: A MODELLING STUDY."

[3] "PROJECTED OUTCOMES OF UNIVERSAL TESTING AND TREATMENT IN A GENERALISED HIV EPIDEMIC IN ZAMBIA AND SOUTH AFRICA (THE HPTN 071 [POPART] TRIAL): A MODELLING STUDY."

[4] "LEVERAGING MODULARITY DURING REPLICATION OF HIGH-FIDELITY MODELS: LESSONS FROM REPLICATING AN AGENT-BASED MODEL FOR HIV PREVENTION."

[5] "EPICE-HIV: AN EPIDEMIOLOGIC COST-EFFECTIVENESS MODEL FOR HIV TREATMENT."

[6] "IMPACT OF THE CENTERS FOR DISEASE CONTROL'S HIV PREEXPOSURE PROPHYLAXIS GUIDELINES FOR MEN WHO HAVE SEX WITH MEN IN THE UNITED STATES."

[7] "PROJECTED HIV AND BACTERIAL SEXUALLY TRANSMITTED INFECTION INCIDENCE FOLLOWING COVID-19-RELATED SEXUAL DISTANCING AND CLINICAL SERVICE INTERRUPTION."
