# Supplementary material for: Modelling HIV/AIDS epidemiological complexity: A scoping review of Agent-Based Models and their application
Source: PLoS One. 2024 Feb 2;19(2):e0297247. doi: 10.1371/journal.pone.0297247 (PMC10836677; doi:10.1371/journal.pone.0297247)
Supplement: S2 Appendix — (DOCX) [file pone.0297247.s002.docx]

**S2 Appendix - Data charting**

We manually identified the region, population and aim in each study following this checklist:

check if the population and region was specified in the title or the abstract.

1. If yes, we registered as presented in the paper.
2. If don’t, we search in the paper if this information exists, and control the result.
3. When no region or population is specified, we identify which was used to calibrate the model.
4. If no information was identified, we specified it as NA.
5. We specify in a new column the region information in the dimension of interest (Country, region, city, neighborhood), and the country of reference (when applicable).

Not Specified - Some studies have no specification of region or population because have other goals and we opted to let without a specification even if some specific (local, or population) information would be used for calibration.

**Region**

We were interested in knowing if the studies were focused on a specific country, city, state, region, or even neighborhood. The challenge is that several studies calibrate their parameters based on empirical measures from specific countries. However, they are not, necessarily, studies focused on a specific region. We define that as, if the paper introduces a specific region in its narrative or research question, we consider this region. If it’s not, we considered as NA.

We specified the location addressed by the study as the variable *Region* and, then, categorizes this information on Geographic Dimension and Country.

**Geographic Dimension**

We categorize the geographical dimension of analysis of each paper in the follow categories:

When a paper deal with more than one geographic dimension we opted for the “larger” dimension (i.e., if deal with cities and states, we categorize as state).

**Table A. Description of Geographic Dimension categorization.**

| **Geographic Dimension** | **Description** |
| --- | --- |
| *Global* | Study focused in a global analysis. |
| *Continent* | Study focused in a specific continent of the globe. |
| *Macro-region* | A region that content more than one country (i.e. sub-Saharan Africa) |
| *Country* | Study focused on one or more countries, individually. |
| *Region* | Study focused on a country region, or a set of states or provinces. |
| *State* | Study focused on one state or province. |
| *Micro-region* | Study focused on a metropolitan area with one or more cities, or other geographical subdivisions inside a *State* dimension. |
| *City* | Study focused on a specifically city, district, municipality, or county. |
| *Neighborhood* | Study focused on one or more neighborhood, community or villages that characterizes small geographical agglomerations. |

**Country**

We record the country of the region of study. This information doesn’t apply to studies that explore macro-regions, such as continents or global studies.

**Population**

We categorize as *Population* the subpopulation (or subpopulations) of interest in the study:

**Table B. Description of Population categorization.**

| **Population** | **Description** |
| --- | --- |
| *Age* | When some Age distribution is of interest of the analysis. |
| *MSWS* | Cisgender Male sex worker. |
| *FSW* | Female Sex Worker. |
| *Heterosexual* | Heterosexual population, or (serodiscordant) couples. |
| *MSM* | Men who have sex with men. Some papers target homosexuals or gay men, we considered as MSM subpopulation. |
| *NIDU* | Non-injection-drug user. |
| *NU* | non-drug user. |
| *PLWH* | People Living with HIV. |
| *PWID* | People who inject drugs. |
| *Race Disparities* | Studies focused on race disparities. We aggregated different subpopulations that explore racial dimensions of the phenomenon. Black/African-American and their subpopulations (MSM, WSW, FSW, Young) are the majority, but also Latin/Hispanic populations are here. |
| *Representative* | When the population it’s not restrict to one specific subpopulation. |
| *WSW* | Women who have sex with women. |
| Young | Adolescents with different age definitions. |
| Women | Focused just on women, or Pregnant, or Vertical Transmission |

**Aims**

We try to synthesize the type of study by its general aim. Some studies are suited to more than one category. We opted to keep booth, or more, when it is the case.

The classification follows these criteria:

**Table 1. Description of aim’s categorization.**

| **Aim** | **Description** |
| --- | --- |
| *ART* | Studies that focused on antiretrovirals. |
| *Cost-Effectiveness* | Studies that aim to evaluate the cost-effectiveness of interventions. |
| *LTC* | When the study is focused on Linkage to Care (LTC) interventions. |
| *Multidisease* | Studies that explore co-infections with other diseases. |
| *PrEP* | Studies that focus on PREP interventions and outcomes |
| *Prevention Packages* | Studies that explore more than one intervention (90-90-90, ART, ART; behavioral intervention to decrease sexual risk behaviors) and/or their combination on HIV/AIDS outcomes |
| *Proof of Concept* | Study that explores some concept or hypothesis by a simulation exercise. |
| *RCT Design* | Studies that applied ABM for the design or discussion of Random Control Trials. |
| *Replication Exercise* | Study that replicates a previous application, or model application without a specific aim. |
| *Single Intervention* | When the study is focused on the effects of only one intervention. Except ART and PrEP that have specific categories. |
| *Social Impact* | Studies that declare as aim the social impacts of HIV/AIDS |
| *Transmission Dynamic* | Study that focused on some feature of the transmission dynamic of HIV/AIDS. *i.e.* epidemiological burning in specific populations such as MSM or Black/African-Americans. |
| *Epidemiologic Analysis* | Studies that aim to predict or analyze some epidemiological measure, such as incidence, or prevalence of HIV. |
| *COVID-19* | Studies that have as background or interest the Pandemic of COVID-19 |
