# Supplementary material for: Modelling HIV/AIDS epidemiological complexity: A scoping review of Agent-Based Models and their application
Source: PLoS One. 2024 Feb 2;19(2):e0297247. doi: 10.1371/journal.pone.0297247 (PMC10836677; doi:10.1371/journal.pone.0297247)
Supplement: S3 Appendix — (DOCX) [file pone.0297247.s003.docx]

**S3 Appendix – Complete Tables**

Here we present additional information about the data collected and the categories description. We have two major classifications: “appearances by study” and “by appearances”. This is because some articles have more than one specific category. This information is expressed on the tables with “appearances by study” where the column name is “Articles”. The overall information about the specific categories is expressed on the tables “by appearances” where we considered the frequency of each category. In these cases, the column name is “Frequency”.

**Table A – Publications by Year**

| **Year** | **Articles** |
| --- | --- |
| 1999 | 1 |
| 2007 | 3 |
| 2008 | 1 |
| 2009 | 2 |
| 2010 | 1 |
| 2011 | 3 |
| 2012 | 7 |
| 2013 | 7 |
| 2014 | 8 |
| 2015 | 11 |
| 2016 | 11 |
| 2017 | 14 |
| 2018 | 15 |
| 2019 | 13 |
| 2020 | 12 |
| 2021 | 18 |
| 2022 | 19 |
| 2023 | 8 |

**Table B - Aims Appearances by Studies**

| **Goal** | **Articles** |
| --- | --- |
| Transmission Dynamics | 37 |
| Single Intervention | 19 |
| PrEP | 17 |
| Cost-Effectiveness | 15 |
| Prevention Packages | 15 |
| Epidemiologic Analysis | 7 |
| Proof of Concept | 7 |
| ART | 6 |
| LTC | 5 |
| Multidisease | 4 |
| Covid-19 | 2 |
| Proof of Concept; Transmission Dynamics | 2 |
| RCT Design | 2 |
| Replication Exercise | 2 |
| Cost-Effectiveness; Prevention Packages | 1 |
| Multidisease; Covid-19 | 1 |
| Multidisease; LTC | 1 |
| Multidisease; PrEP; Transmission Dynamics; Cost-Effectiveness | 1 |
| PrEP; Cost-Effectiveness | 1 |
| PrEP; LTC | 1 |
| Prevalence Estimate | 1 |
| Prevention Packages; Cost-Effectiveness | 1 |
| Proof of Concept; Multidisease | 1 |
| RCT Design; Prevention Packages | 1 |
| Replication Exercise; Covid-19 | 1 |
| Single Intervention; Multidisease | 1 |
| Transmission Dynamics; Multidisease | 1 |
| Transmission Dynamics; Social Impact | 1 |

LTC: Linkage to care. PrEP: pre-exposure prophylaxis. ART: Antiretroviral Treatment. RCT Design: Random Control Trials

**Table C - Aims by Appearances**

| **Goal** | **Frequency** |
| --- | --- |
| Transmission Dynamics | 42 |
| PrEP | 20 |
| Single Intervention | 20 |
| Cost-Effectiveness | 19 |
| Prevention Packages | 18 |
| Multidisease | 10 |
| Proof of Concept | 10 |
| Epidemiologic Analysis | 7 |
| LTC | 7 |
| ART | 6 |
| Covid-19 | 4 |
| RCT Design | 3 |
| Replication Exercise | 3 |
| Prevalence Estimate | 1 |
| Social Impact | 1 |

LTC: Linkage to care. PrEP: pre-exposure prophylaxis. ART: Antiretroviral Treatment. RCT Design: Random Control Trials

**Table D –Population Appearances by Study**

| **Population** | **Articles** |
| --- | --- |
| MSM | 41 |
| Heterosexual | 27 |
| Representative | 20 |
| PWID | 14 |
| PLWH | 10 |
| MSM; Racial Disparities | 5 |
| MSM; Young | 3 |
| MSM; PLWH | 2 |
| Women | 2 |
| Women; Heterosexual | 2 |
| YBMSM; Racial Disparities | 2 |
| Young | 2 |
| Children | 1 |
| FSW | 1 |
| FSW; Heterosexual | 1 |
| Heterosexual; MSM; WSW; PWID; NIDU; NU | 1 |
| Heterosexual; PWID | 1 |
| Heterosexual; Women | 1 |
| MSM; Heterosexual | 1 |
| MSWS | 1 |
| Migrants | 1 |
| PWID; NU; FSW | 1 |
| PWID; Racial Disparities; Incarcerated | 1 |
| Racial Disparities | 1 |
| Racial Disparities; Incarcerated | 1 |
| Women; FSW | 1 |
| Women; FSW; MSM; Heterosexual | 1 |
| YMSM | 1 |
| YMSM; Racial Disparities | 1 |
| Young; Racial Disparities; MSM | 1 |

PLWH: People Living with HIV. MSM: Men who have sex with men. FSW: Female sex workers. PWID: People who inject drugs. WSW: women who have sex with women. NU: non-drug users. NIDU: non-injection-drug.

**Table E –Population by Appearances**

| **Population** | **Frequency** |
| --- | --- |
| MSM | 55 |
| Heterosexual | 35 |
| Representative | 20 |
| PWID | 18 |
| PLWH | 12 |
| Racial Disparities | 12 |
| Women | 7 |
| Young | 6 |
| FSW | 5 |
| Incarcerated | 2 |
| NU | 2 |
| YBMSM | 2 |
| YMSM | 2 |
| Children | 1 |
| Migrants | 1 |
| MSWS | 1 |
| NIDU | 1 |
| WSW | 1 |

PLWH: People Living with HIV. MSM: Men who have sex with men. FSW: Female sex workers. PWID: People who inject drugs. WSW: women who have sex with women. NU: non-drug users. NIDU: non-inject drug users.

**Table F. Locations by Study**

| **Location** | **Articles** |
| --- | --- |
| South Africa | 15 |
| US | 15 |
| Sub-Saharan Africa | 8 |
| New York City, NY - US | 6 |
| UK | 6 |
| Atlanta, GA - US | 5 |
| Netherlands | 5 |
| Not Specified | 5 |
| NA | 5 |
| Atlanta, GA, US | 4 |
| Kenya | 4 |
| Kwazulu-Natal, South Africa | 4 |
| New South Wales - Australia | 3 |
| Rhode Island - US | 3 |
| Scott County, Indiana, US | 3 |
| Uganda | 3 |
| Zambia; South Africa | 3 |
| Amsterdam, Netherlands | 2 |
| Atlanta, GA - US; Seatle, WA - US | 2 |
| Atlanta, Sandy Springs, Alpharetta, GA - US | 2 |
| Baltimore City, Mariland - US | 2 |
| Illinois - US | 2 |
| Malawi | 2 |
| Manicaland, Zimbabwe | 2 |
| Philadelphia, PE - US | 2 |
| Victoria - Australia | 2 |
| Bushwick, Brooklyn, NY - US | 1 |
| Africa | 1 |
| Algeria; Bahrain; Djibouti; Iran; Libya; Morocco; Pakistan; Somalia; South Sudan; Sudan; Tunisia; Yemen | 1 |
| Athens, Grece | 1 |
| Atlanta, Georgia, US | 1 |
| Australia | 1 |
| Baltimore City, Maryland, US | 1 |
| Botswana | 1 |
| Cabo Verde | 1 |
| Cebu City, Vissaias Centrais, Fillipinas | 1 |
| Chicago, Illinois | 1 |
| Cotonou, Benin; Yaounde, Cameroon; Kisumu, Kenya; Ndola, Zambia | 1 |
| Delaware, Florida, Hawaii, Illinois, Michigan, New York City, Rhode Island, San Diego | 1 |
| Durban - South Africa | 1 |
| Eswantini | 1 |
| France | 1 |
| Global | 1 |
| Houston, Texas, US | 1 |
| Kenya; Zimbabwe; South Africa | 1 |
| Kisumu, Kenya; Ndola, Zambia; Yaounde, Cameroon; Cotonou, Benin | 1 |
| Kwazulu-Natal - South Africa; Southwestern, Uganda | 1 |
| Latvia | 1 |
| Melbourne - Australia | 1 |
| Memphis, TN - US | 1 |
| Mississippi | 1 |
| Montreal, Canada | 1 |
| NY Metropolitan Statistical Area (MSA), NY - US | 1 |
| Nyanza, Kenya | 1 |
| Rakai, Uganda | 1 |
| Rural Indiana - US | 1 |
| Sekhukhune, Limpopo - South Africa | 1 |
| South Africa; Kenya | 1 |
| South, US | 1 |
| Sweden | 1 |
| Taiwan | 1 |
| Thailand | 1 |
| Uganda; Kenya | 1 |
| Western Kenya | 1 |
| Zambia | 1 |

**Table G. Locations by Appearance**

| **Location** | **Frequency** |
| --- | --- |
| South Africa | 20 |
| US | 15 |
| Sub-Saharan Africa | 8 |
| Atlanta, GA - US | 7 |
| Kenya | 7 |
| New York City, NY - US | 6 |
| UK | 6 |
| Netherlands | 5 |
| Not Specified | 5 |
| Atlanta, GA, US | 4 |
| Kwazulu-Natal, South Africa | 4 |
| Uganda | 4 |
| Zambia | 4 |
| New South Wales - Australia | 3 |
| Rhode Island - US | 3 |
| Scott County, Indiana, US | 3 |
| Amsterdam, Netherlands | 2 |
| Atlanta, Sandy Springs, Alpharetta, GA - US | 2 |
| Baltimore City, Mariland - US | 2 |
| Cotonou, Benin | 2 |
| Illinois - US | 2 |
| Kisumu, Kenya | 2 |
| Malawi | 2 |
| Manicaland, Zimbabwe | 2 |
| Ndola, Zambia | 2 |
| Philadelphia, PE - US | 2 |
| Seatle, WA - US | 2 |
| Victoria - Australia | 2 |
| Yaounde, Cameroon | 2 |
| Bushwick, Brooklyn, NY - US | 1 |
| Africa | 1 |
| Algeria | 1 |
| Athens, Grece | 1 |
| Atlanta, Georgia, US | 1 |
| Australia | 1 |
| Bahrain | 1 |
| Baltimore City, Maryland, US | 1 |
| Botswana | 1 |
| Cabo Verde | 1 |
| Cebu City, Vissaias Centrais, Fillipinas | 1 |
| Chicago, Illinois | 1 |
| Delaware, Florida, Hawaii, Illinois, Michigan, New York City, Rhode Island, San Diego | 1 |
| Djibouti | 1 |
| Durban - South Africa | 1 |
| Eswantini | 1 |
| France | 1 |
| Global | 1 |
| Houston, Texas, US | 1 |
| Iran | 1 |
| Kwazulu-Natal - South Africa | 1 |
| Latvia | 1 |
| Libya | 1 |
| Melbourne - Australia | 1 |
| Memphis, TN - US | 1 |
| Mississippi | 1 |
| Montreal, Canada | 1 |
| Morocco | 1 |
| NY Metropolitan Statistical Area (MSA), NY - US | 1 |
| Nyanza, Kenya | 1 |
| Pakistan | 1 |
| Rakai, Uganda | 1 |
| Rural Indiana - US | 1 |
| Sekhukhune, Limpopo - South Africa | 1 |
| Somalia | 1 |
| South Sudan | 1 |
| South, US | 1 |
| Southwestern, Uganda | 1 |
| Sudan | 1 |
| Sweden | 1 |
| Taiwan | 1 |
| Thailand | 1 |
| Tunisia | 1 |
| Western Kenya | 1 |
| Yemen | 1 |
| Zimbabwe | 1 |

**Table H. Geographic Dimensions by Appearances**

| **Geographic Dimension** | **Articles** |
| --- | --- |
| Country | 62 |
| City | 40 |
| State | 19 |
| Macro-region | 13 |
| Micro-Region | 4 |
| Region | 3 |
| Continent | 1 |
| Global | 1 |
| Neighborhood | 1 |

**Table I. Countries Appearances by Study**

| **Country** | **Frequency** |
| --- | --- |
| US | 57 |
| South Africa | 27 |
| Kenya | 11 |
| Australia | 7 |
| Netherlands | 7 |
| Uganda | 6 |
| UK | 6 |
| Zambia | 6 |
| Zimbabwe | 3 |
| Benin | 2 |
| Camerron | 2 |
| Malawi | 2 |
| Botswana | 1 |
| Cabo Verde | 1 |
| Canada | 1 |
| Eswantini | 1 |
| Fillipinas | 1 |
| France | 1 |
| Greece | 1 |
| Latvia | 1 |
| Sweden | 1 |
| Taiwan | 1 |
| Thailand | 1 |

**Table J – Use of ODD protocol and declaration of Limitations into the article**

| **ODD** | **Limitations** | **Articles** |
| --- | --- | --- |
| No | No | 32 |
| No | Yes | 107 |
| No | NA | 0 |
| Yes | No | 2 |
| Yes | Yes | 4 |
| NA | No | 1 |
| NA | NA | 8 |

ODD: Overview, Design Concepts and Details protocol suggested by Grimm and colleagues for results’ presentation for Individual Based Methods (IBM). Limitations: If the article express, explicitly, its limitations. NA: We couldn’t get full access to the articles.
