# Supplementary material for: Modelling HIV/AIDS epidemiological complexity: A scoping review of Agent-Based Models and their application
Source: PLoS One. 2024 Feb 2;19(2):e0297247. doi: 10.1371/journal.pone.0297247 (PMC10836677; doi:10.1371/journal.pone.0297247)
Supplement: S4 Appendix — (PDF) [file pone.0297247.s004.pdf]

S4 Appendix - PRISMA Checklist

TITLE
Title - Identify the report as a systematic review, meta-analysis, or both.
Page 1, line 1.

ABSTRACT
Structured summary - Provide a structured summary including, as applicable: background; objectives; data sources; study eligibility criteria, participants, and interventions; study appraisal and synthesis methods; results; limitations; conclusions and implications of key findings; systematic review registration number.
Page 2

INTRODUCTION
Rationale - Describe the rationale for the review in the context of what is already known.
Page 3
Objectives - Provide an explicit statement of questions being addressed with reference to participants, interventions, comparisons, outcomes, and study design (PICOS).
Page 4

METHODS
Protocol and registration - Indicate if a review protocol exists, if and where it can be accessed (e.g., Web address), and, if available, provide registration information including registration number.
Not applicable for scoping review

Eligibility criteria - Specify study characteristics (e.g., PICOS, length of follow-up) and report characteristics (e.g., years considered, language, publication status) used as criteria for eligibility, giving rationale.
Page 4-5

Information sources - Describe all information sources (e.g., databases with dates of coverage, contact with study authors to identify additional studies) in the search and date last searched.
Page 4

Search - Present full electronic search strategy for at least one database, including any limits used, such that it could be repeated.
Page 4 and S1 Appendix.

Study selection - State the process for selecting studies (i.e., screening, eligibility, included in systematic review, and, if applicable, included in the meta-analysis).
Page 4, S1 Appendix and S2 Appendix

Data collection process - Describe method of data extraction from reports (e.g., piloted forms, independently, in duplicate) and any processes for obtaining and confirming data from investigators.
Page 4-5

Data items - List and define all variables for which data were sought (e.g., PICOS, funding sources) and any assumptions and simplifications made.
Page 4-5 and S2 Appendix

Risk of bias in individual studies - Describe methods used for assessing risk of bias of individual studies (including specification of whether this was done at the study or outcome level), and how this information is to be used in any data synthesis.
Page 5

Summary measures - State the principal summary measures (e.g., risk ratio, difference in means).
Not applicable for scoping reviews.

Synthesis of results - Describe the methods of handling data and combining results of studies, if done, including measures of consistency (e.g., I2 for each meta-analysis).

Page 6 and S2 Appendix

Risk of bias across studies - Specify any assessment of risk of bias that may affect the cumulative evidence (e.g., publication bias, selective reporting within studies).
Not applicable for scoping reviews.

Additional analyses - Describe methods of additional analyses (e.g., sensitivity or subgroup analyses, meta-regression), if done, indicating which were pre-specified.
Not applicable for scoping reviews.

RESULTS
Study selection - Give numbers of studies screened, assessed for eligibility, and included in the review, with reasons for exclusions at each stage, ideally with a flow diagram.
Page 6, Figure 2.

Study characteristics - For each study, present characteristics for which data were extracted (e.g., study size, PICOS, follow-up period) and provide the citations.
Page 6

Risk of bias within studies - Present data on risk of bias of each study and, if available, any outcome level assessment (see item 12).
Not applicable for scoping reviews.

Results of individual studies - For all outcomes considered (benefits or harms), present, for each study: (a) simple summary data for each intervention group (b) effect estimates and confidence intervals, ideally with a forest plot.
Not applicable for scoping reviews.

Synthesis of results - Present results of each meta-analysis done, including confidence intervals and measures of consistency.
Not applicable for scoping reviews.

Risk of bias across studies - Present results of any assessment of risk of bias across studies.
Not applicable for scoping reviews.

Additional analysis - Give results of additional analyses, if done (e.g., sensitivity or subgroup analyses, meta-regression).
Not applicable for scoping reviews.

DISCUSSION
Summary of evidence - Summarize the main findings including the strength of evidence for each main outcome; consider their relevance to key groups (e.g., healthcare providers, users, and policy makers).
Page 12

Limitations - Discuss limitations at study and outcome level (e.g., risk of bias), and at review-level (e.g., incomplete retrieval of identified research, reporting bias).
Page 14

Conclusions - Provide a general interpretation of the results in the context of other evidence, and implications for future research.
Page 14-15

FUNDING

Funding - Describe sources of funding for the systematic review and other support (e.g., supply of data); role of funders for the systematic review.
Page 15
