# Supplementary material for: Modelling HIV/AIDS epidemiological complexity: A scoping review of Agent-Based Models and their application
Source: PLoS One. 2024 Feb 2;19(2):e0297247. doi: 10.1371/journal.pone.0297247 (PMC10836677; doi:10.1371/journal.pone.0297247)
Supplement: S1 Table — (DOCX) [file pone.0297247.s006.docx]

**S5 Table – Studies dataset with charted variables**

| **Study** | **Title** | **Aim** | **Population** | **Location** | **Geographic Dimension** | **Country** | **ODD** | **Limitations** |
| --- | --- | --- | --- | --- | --- | --- | --- | --- |
| NEAIGUS A, 1999, PUBLIC HEALTH | THE NETWORK APPROACH AND INTERVENTIONS TO PREVENT HIV AMONG INJECTION DRUG USERS | Transmission Dynamics | PWID | NA | NA | NA | NA | No |
| ALAM SJ, 2007, JASSS | THE IMPACT OF HIV/AIDS IN THE CONTEXT OF SOCIOECONOMIC STRESSORS: AN EVIDENCE-DRIVEN APPROACH | Transmission Dynamics; Social Impact | Representative | Sekhukhune, Limpopo - South Africa | City | South Africa | No | Yes |
| FREEMAN EE, 2007, SEX TRANSM INFECT | PROPORTION OF NEW HIV INFECTIONS ATTRIBUTABLE TO HERPES SIMPLEX 2 INCREASES OVER TIME: SIMULATIONS OF THE CHANGING ROLE OF SEXUALLY TRANSMITTED INFECTIONS IN SUB-SAHARAN AFRICAN HIV EPIDEMICS | Transmission Dynamics; Multidisease | Representative | Kisumu, Kenya; Ndola, Zambia; Yaounde, Cameroon; Cotonou, Benin | City | Kenya; Zambia; Camerron; Benin | No | Yes |
| ORROTH KK, 2007, SEX TRANSM INFECT | UNDERSTANDING THE DIFFERENCES BETWEEN CONTRASTING HIV EPIDEMICS IN EAST AND WEST AFRICA: RESULTS FROM A SIMULATION MODEL OF THE FOUR CITIES STUDY | Transmission Dynamics | Heterosexual | Cotonou, Benin; Yaounde, Cameroon; Kisumu, Kenya; Ndola, Zambia | City | Benin; Camerron; Kenya; Zambia | No | Yes |
| WHITE RG, 2008, AIDS | MALE CIRCUMCISION FOR HIV PREVENTION IN SUB-SAHARAN AFRICA: WHO, WHAT AND WHEN? | Single Intervention | Heterosexual | Sub-Saharan Africa | Macro-region | NA | No | No |
| FREEMAN EE, 2009, VACCINE | POPULATION-LEVEL EFFECT OF POTENTIAL HSV2 PROPHYLACTIC VACCINES ON HIV INCIDENCE IN SUB-SAHARAN AFRICA | Single Intervention | Representative | Sub-Saharan Africa | Macro-region | NA | No | No |
| LECLERC PM, 2009, PLOS ONE | FITTING THE HIV EPIDEMIC IN ZAMBIA: A TWO-SEX MICRO-SIMULATION MODEL | Transmission Dynamics | Heterosexual; Women | Zambia | Country | Zambia | No | Yes |
| KIM JH, 2010, EPIDEMIOLOGY | HIV TRANSMISSION BY STAGE OF INFECTION AND PATTERN OF SEXUAL PARTNERSHIPS | Transmission Dynamics | Heterosexual | NA | NA | NA | No | No |
| GRAY RT, 2011, VACCINE | EXPECTED EPIDEMIOLOGICAL IMPACT OF THE INTRODUCTION OF A PARTIALLY EFFECTIVE HIV VACCINE AMONG MEN WHO HAVE SEX WITH MEN IN AUSTRALIA | Single Intervention | MSM | New South Wales - Australia | State | Australia | NA | NA |
| WILSON DP, 2011, SEX TRANSM INFECT | REPLACEMENT OF CONVENTIONAL HIV TESTING WITH RAPID TESTING: MATHEMATICAL MODELLING TO PREDICT THE IMPACT ON FURTHER HIV TRANSMISSION BETWEEN MEN | Single Intervention | MSM | Melbourne - Australia | City | Australia | No | Yes |
| SCHNEIDER K, 2011, AIDS | ECONOMIC EVALUATION OF MONITORING VIROLOGIC RESPONSES TO ANTIRETROVIRAL THERAPY IN HIV-INFECTED CHILDREN IN RESOURCE-LIMITED SETTINGS | ART | Children | Thailand | Country | Thailand | No | Yes |
| GRAW F, 2012, EPIDEMICS | AGENT-BASED AND PHYLOGENETIC ANALYSES REVEAL HOW HIV-1 MOVES BETWEEN RISK GROUPS: INJECTING DRUG USERS SUSTAIN THE HETEROSEXUAL EPIDEMIC IN LATVIA | Transmission Dynamics | Heterosexual; PWID | Latvia | Country | Latvia | No | No |
| RICHARDSON L, 2012, SOC SCI RES | MODELING THE IMPACT OF SUPRA-STRUCTURAL NETWORK NODES: THE CASE OF ANONYMOUS SYRINGE SHARING AND HIV AMONG PEOPLE WHO INJECT DRUGS | Transmission Dynamics | PWID | Bushwick, Brooklyn, NY - US | Neighborhood | US | No | Yes |
| MARSHALL BDL, 2012, PLOS ONE | A COMPLEX SYSTEMS APPROACH TO EVALUATE HIV PREVENTION IN METROPOLITAN AREAS: PRELIMINARY IMPLICATIONS FOR COMBINATION INTERVENTION STRATEGIES | Prevention Packages | Heterosexual; MSM; WSW; PWID; NIDU; NU | NY Metropolitan Statistical Area (MSA), NY - US | Micro-Region | US | Yes | Yes |
| BEYRER C, 2012, LANCET | GLOBAL EPIDEMIOLOGY OF HIV INFECTION IN MEN WHO HAVE SEX WITH MEN | Transmission Dynamics | MSM | Global | Global |  | No | No |
| JANSSON J, 2012, PLOS ONE | PROJECTED DEMOGRAPHIC PROFILE OF PEOPLE LIVING WITH HIV IN AUSTRALIA: PLANNING FOR AN OLDER GENERATION | ART | PLWH | Australia | Country | Australia | No | Yes |
| HOARE A, 2012, SEX HEALTH | COULD IMPLEMENTATION OF AUSTRALIA'S NATIONAL GAY MEN'S SYPHILIS ACTION PLAN HAVE AN INDIRECT EFFECT ON THE HIV EPIDEMIC? | Multidisease | MSM | Victoria - Australia | State | Australia | NA | NA |
| MCCREESH N, 2012, SEX TRANSM DIS | EXPLORING THE POTENTIAL IMPACT OF A REDUCTION IN PARTNERSHIP CONCURRENCY ON HIV INCIDENCE IN RURAL UGANDA: A MODELING STUDY | Single Intervention | Representative | Uganda | Country | Uganda | No | Yes |
| TULLY S, 2013, J THEOR BIOL | COEVOLUTION OF RISK PERCEPTION, SEXUAL BEHAVIOUR, AND HIV TRANSMISSION IN AN AGENT-BASED MODEL | Transmission Dynamics | MSM | Not Specified | NA | NA | No | No |
| ROMERO-SEVERSON EO, 2013, EPIDEMIOLOGY | ACUTE-STAGE TRANSMISSION OF HIV: EFFECT OF VOLATILE CONTACT RATES | Transmission Dynamics | NA | NA | NA | NA | No | No |
| GRAY RT, 2013, PLOS ONE | INCREASED HIV TESTING WILL MODESTLY REDUCE HIV INCIDENCE AMONG GAY MEN IN NSW AND WOULD BE ACCEPTABLE IF HIV TESTING BECOMES CONVENIENT | Single Intervention | MSM | New South Wales - Australia | State | Australia | No | Yes |
| DE VOS AS, 2013, ADDICTION | DECLINE IN INCIDENCE OF HIV AND HEPATITIS C VIRUS INFECTION AMONG INJECTING DRUG USERS IN AMSTERDAM; EVIDENCE FOR HARM REDUCTION? | Epidemiologic Analysis | PWID | Amsterdam, Netherlands | City | Netherlands | NA | NA |
| PHILLIPS AN, 2013, PLOS ONE | INCREASED HIV INCIDENCE IN MEN WHO HAVE SEX WITH MEN DESPITE HIGH LEVELS OF ART-INDUCED VIRAL SUPPRESSION: ANALYSIS OF AN EXTENSIVELY DOCUMENTED EPIDEMIC | ART | MSM | UK | Country | UK | No | No |
| WOHL DA, 2013, Antimicrob. AGENTS Chemother. | RALTEGRAVIR PHARMACOKINETICS IN TREATMENT-NAIVE PATIENTS IS NOT INFLUENCED BY RACE: RESULTS FROM THE RALTEGRAVIR EARLY THERAPY IN AFRICAN-AMERICANS LIVING WITH HIV (REAL) STUDY | Transmission Dynamics | Heterosexual | South Africa | Country | South Africa | No | Yes |
| NG KY, 2013, BMC Infect. Dis. | HIGH PREVALENCE OF CXCR4 USAGE AMONG TREATMENT-NAIVE CRF01_AE AND CRF51_01B-INFECTED HIV-1 SUBJECTS IN SINGAPORE | Prevention Packages | MSM | South Africa | Country | South Africa | No | No |
| CUADROS DF, 2014, COMPUT BIOL MED | USE OF AGENT-BASED SIMULATIONS TO DESIGN AND INTERPRET HIV CLINICAL TRIALS | RCT Design; Prevention Packages | Heterosexual | Rakai, Uganda | City | Uganda | No | Yes |
| BROOKMEYER R, 2014, PLOS ONE | COMBINATION HIV PREVENTION AMONG MSM IN SOUTH AFRICA: RESULTS FROM AGENT-BASED MODELING | Prevention Packages | MSM | South Africa | Country | South Africa | No | Yes |
| WHITE PJ, 2014, J INFECT DIS | HOW MANY HIV INFECTIONS MAY BE AVERTED BY TARGETING PRIMARY INFECTION IN MEN WHO HAVE SEX WITH MEN? QUANTIFICATION OF CHANGES IN TRANSMISSION-RISK BEHAVIOR, USING AN INDIVIDUAL-BASED MODEL | Transmission Dynamics | MSM | UK | Country | UK | No | Yes |
| BOREN D, 2014, STAT MED | STOCHASTIC VARIATION IN NETWORK EPIDEMIC MODELS: IMPLICATIONS FOR THE DESIGN OF COMMUNITY LEVEL HIV PREVENTION TRIALS | Transmission Dynamics | MSM | South Africa | Country | South Africa | No | No |
| SCHNEIDER K, 2014, CLIN INFECT DIS | A COST-EFFECTIVENESS ANALYSIS OF HIV PREEXPOSURE PROPHYLAXIS FOR MEN WHO HAVE SEX WITH MEN IN AUSTRALIA | Cost-Effectiveness | MSM | New South Wales - Australia | State | Australia | No | Yes |
| HERBECK JT, 2014, PLOS COMPUT BIOL | AN HIV EPIDEMIC MODEL BASED ON VIRAL LOAD DYNAMICS: VALUE IN ASSESSING EMPIRICAL TRENDS IN HIV VIRULENCE AND COMMUNITY VIRAL LOAD | Transmission Dynamics | NA | Not Specified | NA | NA | No | No |
| WANG R, 2014, CLIN TRIALS | SAMPLE SIZE CONSIDERATIONS IN THE DESIGN OF CLUSTER RANDOMIZED TRIALS OF COMBINATION HIV PREVENTION | RCT Design | Heterosexual | Botswana | Country | Botswana | No | Yes |
| MCCORMICK AW, 2014, PLOS ONE | DEVELOPMENT, CALIBRATION AND PERFORMANCE OF AN HIV TRANSMISSION MODEL INCORPORATING NATURAL HISTORY AND BEHAVIORAL PATTERNS: APPLICATION IN SOUTH AFRICA | Replication Exercise | Representative | South Africa | Country | South Africa | No | Yes |
| HUANG CY, 2015, COMPUT MATH METHOD MED | AN AGENT-BASED EPIDEMIC SIMULATION OF SOCIAL BEHAVIORS AFFECTING HIV TRANSMISSION AMONG TAIWANESE HOMOSEXUALS | Transmission Dynamics | MSM | Taiwan | Country | Taiwan | No | No |
| RENIERS G, 2015, DEMOGR RES | SEXUAL NETWORKS, PARTNERSHIP MIXING, AND THE FEMALE-TO-MALE RATIO OF HIV INFECTIONS IN GENERALIZED EPIDEMICS: AN AGENT-BASED SIMULATION STUDY | Transmission Dynamics | Heterosexual | Africa | Continent | NA | No | No |
| MONTEIRO JFG, 2015, INT J PUBLIC HEALTH | EVALUATING HIV PREVENTION STRATEGIES FOR POPULATIONS IN KEY AFFECTED GROUPS: THE EXAMPLE OF CABO VERDE | Prevention Packages | PWID; NU; FSW | Cabo Verde | Country | Cabo Verde | No | Yes |
| TULLY S, 2015, SCI REP | SEXUAL BEHAVIOR, RISK PERCEPTION, AND HIV TRANSMISSION CAN RESPOND TO HIV ANTIVIRAL DRUGS AND VACCINES THROUGH MULTIPLE PATHWAYS | Transmission Dynamics | MSM; Young | Not Specified | NA | NA | No | No |
| BECK EC, 2015, JAIDS | A DATA-DRIVEN SIMULATION OF HIV SPREAD AMONG YOUNG MEN WHO HAVE SEX WITH MEN: ROLE OF AGE AND RACE MIXING AND STIS | Transmission Dynamics | YMSM; Racial Disparities | US | Country | US | No | Yes |
| KLEIN DJ, 2015, INT HEALTH | TARGETING HIV SERVICES TO MALE MIGRANT WORKERS IN SOUTHERN AFRICA WOULD NOT REVERSE GENERALIZED HIV EPIDEMICS IN THEIR HOME COMMUNITIES: A MATHEMATICAL MODELING ANALYSIS | Single Intervention | Heterosexual | Sub-Saharan Africa | Macro-region | NA | No | No |
| PHILLIPS AN, 2015, AIDS | POTENTIAL IMPACT ON HIV INCIDENCE OF HIGHER HIV TESTING RATES AND EARLIER ANTIRETROVIRAL THERAPY INITIATION IN MSM | Single Intervention | MSM | UK | Country | UK | No | No |
| DIMITROV D, 2015, PLOS ONE | HIGH INCIDENCE IS NOT HIGH EXPOSURE: WHAT PROPORTION OF PREVENTION TRIAL PARTICIPANTS ARE EXPOSED TO HIV? | Transmission Dynamics | Women; Heterosexual | South Africa | Country | South Africa | No | No |
| SMITH JA, 2015, LANCET HIV | COST-EFF ECTIVENESS OF COMMUNITY-BASED STRATEGIES TO STRENGTHEN THE CONTINUUM OF HIV CARE IN RURAL SOUTH AFRICA: A HEALTH ECONOMIC MODELLING ANALYSIS | Cost-Effectiveness; Prevention Packages | Heterosexual | Kwazulu-Natal, South Africa | State | South Africa | No | No |
| SMIT M, 2015, LANCET INFECT DIS | FUTURE CHALLENGES FOR CLINICAL CARE OF AN AGEING POPULATION INFECTED WITH HIV: A MODELLING STUDY | Multidisease; LTC | PLWH | Netherlands | Country | Netherlands | No | Yes |
| VELEZ JD, 2015, RECOLETOS Multidiscip. Res. J. | A PREDICTIVE MODEL ON THE SPREAD OF HIV IN CEBU CITY | LTC | Representative | Manicaland, Zimbabwe | State | Zimbabwe | No | No |
| MONTEIRO JFG, 2016, EPIDEMIOL INFECT | UNDERSTANDING THE EFFECTS OF DIFFERENT HIV TRANSMISSION MODELS IN INDIVIDUAL-BASED MICROSIMULATION OF HIV EPIDEMIC DYNAMICS IN PEOPLE WHO INJECT DRUGS | Transmission Dynamics | PWID | New York City, NY - US | City | US | No | Yes |
| NAKAGAWA F, 2016, EPIDEMIOLOGY | A METHOD TO ESTIMATE THE SIZE AND CHARACTERISTICS OF HIV-POSITIVE POPULATIONS USING AN INDIVIDUAL-BASED STOCHASTIC SIMULATION MODEL | Prevalence Estimate | MSM | UK | Country | UK | No | Yes |
| ABUELEZAM NN, 2016, AM J EPIDEMIOL | CAN THE HETEROSEXUAL HIV EPIDEMIC BE ELIMINATED IN SOUTH AFRICA USING COMBINATION PREVENTION? A MODELING ANALYSIS | Prevention Packages | Heterosexual | South Africa | Country | South Africa | No | Yes |
| ROBERTS ST, 2016, J INT AIDS SOC | ESTIMATING THE IMPACT OF UNIVERSAL ANTIRETROVIRAL THERAPY FOR HIV SERODISCORDANT COUPLES THROUGH HOME HIV TESTING: INSIGHTS FROM MATHEMATICAL MODELS | Single Intervention | Heterosexual | Kwazulu-Natal - South Africa; Southwestern, Uganda | Region | South Africa; Uganda | No | Yes |
| ESCUDERO DJ, 2016, AIDS | ACUTE HIV INFECTION TRANSMISSION AMONG PEOPLE WHO INJECT DRUGS IN A MATURE EPIDEMIC SETTING | Transmission Dynamics | PWID | New York City, NY - US | City | US | No | Yes |
| HERBECK JT, 2016, VIRUS EVOL | EVOLUTION OF HIV VIRULENCE IN RESPONSE TO WIDESPREAD SCALE UP OF ANTIRETROVIRAL THERAPY: A MODELING STUDY | ART | Heterosexual | South Africa | Country | South Africa | No | No |
| BERSHTEYN A, 2016, INT HEALTH | AGE-TARGETED HIV TREATMENT AND PRIMARY PREVENTION AS A `RING FENCE' TO EFFICIENTLY INTERRUPT THE AGE PATTERNS OF TRANSMISSION IN GENERALIZED EPIDEMIC SETTINGS IN SOUTH AFRICA | Single Intervention | Heterosexual | Kwazulu-Natal, South Africa | State | South Africa | No | Yes |
| TWEYA H, 2016, AIDS | COMPARATIVE COST-EFFECTIVENESS OF OPTION BR FOR PREVENTION OF MOTHER-TO-CHILD TRANSMISSION OF HIV IN MALAWI | Cost-Effectiveness | Women | Malawi | Country | Malawi | No | Yes |
| OLNEY JJ, 2016, LANCET HIV | EVALUATING STRATEGIES TO IMPROVE HIV CARE OUTCOMES IN KENYA: A MODELLING STUDY | Prevention Packages | Representative | Kenya | Country | Kenya | No | No |
| VANDEWALLE B, 2016, PLOS ONE | EPICE-HIV: AN EPIDEMIOLOGIC COST-EFFECTIVENESS MODEL FOR HIV TREATMENT. | Cost-Effectiveness |  | Not Specified |  |  | No | No |
| JENNESS SM, 2016, J INFECT DIS | IMPACT OF THE CENTERS FOR DISEASE CONTROL'S HIV PREEXPOSURE PROPHYLAXIS GUIDELINES FOR MEN WHO HAVE SEX WITH MEN IN THE UNITED STATES. | PrEP; LTC | MSM | Atlanta, GA, US | City | US | No | Yes |
| GOPALAPPA C, 2017, AIDS | COMBINATIONS OF INTERVENTIONS TO ACHIEVE A NATIONAL HIV INCIDENCE REDUCTION GOAL: INSIGHTS FROM AN AGENT-BASED MODEL | Prevention Packages | PLWH | US | Country | US | No | Yes |
| GOPALAPPA C, 2017, MED DECIS MAK | PROGRESSION AND TRANSMISSION OF HIV/AIDS (PATH 2.0): A NEW, AGENT-BASED MODEL TO ESTIMATE HIV TRANSMISSIONS IN THE UNITED STATES | Transmission Dynamics | Representative | US | Country | US | No | No |
| SAWERS L, 2017, AJAR-AFR J AIDS RES | PARTNERSHIP DURATION, CONCURRENCY, AND HIV IN SUB-SAHARAN AFRICA | Transmission Dynamics | Heterosexual | Sub-Saharan Africa | Macro-region | NA | NA | NA |
| DOMBROWSKI K, 2017, AIDS BEHAV | THE INTERACTION OF RISK NETWORK STRUCTURES AND VIRUS NATURAL HISTORY IN THE NON-SPREADING OF HIV AMONG PEOPLE WHO INJECT DRUGS IN THE EARLY STAGES OF THE EPIDEMIC | Transmission Dynamics | PWID | New York City, NY - US | City | US | No | Yes |
| GOODREAU SM, 2017, LANCET HIV | SOURCES OF RACIAL DISPARITIES IN HIV PREVALENCE IN MEN WHO HAVE SEX WITH MEN IN ATLANTA, GA, USA: A MODELLING STUDY | Transmission Dynamics | MSM; Racial Disparities | Atlanta, GA - US | City | US | No | No |
| ESCUDERO DJ, 2017, BMC PUBLIC HEALTH | THE RISK OF HIV TRANSMISSION AT EACH STEP OF THE HIV CARE CONTINUUM AMONG PEOPLE WHO INJECT DRUGS: A MODELING STUDY | Transmission Dynamics | PWID | New York City, NY - US | City | US | No | Yes |
| SMITH JA, 2017, CONTRACEPTION | COULD MISREPORTING OF CONDOM USE EXPLAIN THE OBSERVED ASSOCIATION BETWEEN INJECTABLE HORMONAL CONTRACEPTIVES AND HIV ACQUISITION RISK | Proof of Concept | Heterosexual | South Africa | Country | South Africa | No | Yes |
| OMORI R, 2017, AIDS | SEXUAL NETWORK DRIVERS OF HIV AND HERPES SIMPLEX VIRUS TYPE 2 TRANSMISSION | Proof of Concept; Transmission Dynamics | NA | NA | NA | NA | No | Yes |
| NAKAGAWA F, 2017, AIDS | AN EPIDEMIOLOGICAL MODELLING STUDY TO ESTIMATE THE COMPOSITION OF HIV-POSITIVE POPULATIONS INCLUDING MIGRANTS FROM ENDEMIC SETTINGS | Transmission Dynamics | Migrants | UK | Country | UK | No | Yes |
| MCCREESH N, 2017, BMC INFECT DIS | UNIVERSAL TEST, TREAT, AND KEEP: IMPROVING ART RETENTION IS KEY IN COST-EFFECTIVE HIV CONTROL IN UGANDA | Prevention Packages | Representative | Uganda | Country | Uganda | No | Yes |
| KASAIE P, 2017, JAIDS | THE IMPACT OF PREEXPOSURE PROPHYLAXIS AMONG MEN WHO HAVE SEX WITH MEN: AN INDIVIDUAL-BASED MODEL | PrEP | MSM | Baltimore City, Maryland, US | City | US | No | Yes |
| JENNESS SM, 2017, PLOS ONE | INDIVIDUAL HIV RISK VERSUS POPULATION IMPACT OF RISK COMPENSATION AFTER HIV PREEXPOSURE PROPHYLAXIS INITIATION AMONG MEN WHO HAVE SEX WITH MEN | PrEP | MSM | Atlanta, GA, US | City | US | No | Yes |
| RENTZ MF, 2017, Curr. HIV Res. | PILOT INTEGRATION OF HIV SCREENING AND HEALTHCARE SETTINGS WITH MULTI- COMPONENT SOCIAL NETWORK AND PARTNER TESTING FOR HIV DETECTION | Epidemiologic Analysis | Heterosexual | Cebu City, Vissaias Centrais, Fillipinas | City | Fillipinas | No | No |
| RAGONNET R, 2017, OPEN Forum Infect. Dis. | ESTIMATING THE TIME TO DIAGNOSIS AND THE CHANCE OF SPONTANEOUS CLEARANCE DURING ACUTE HEPATITIS C IN HUMAN IMMUNODEFICIENCY VIRUS-INFECTED INDIVIDUALS | Transmission Dynamics | NA | New York City, NY - US | City | US | No | Yes |
| KASAIE P, 2018, SEX TRANSM DIS | IMPACT OF PROVIDING PREEXPOSURE PROPHYLAXIS FOR HUMAN IMMUNODEFICIENCY VIRUS AT CLINICS FOR SEXUALLY TRANSMITTED INFECTIONS IN BALTIMORE CITY: AN AGENT-BASED MODEL | PrEP | MSM | Baltimore City, Mariland - US | City | US | No | Yes |
| ADAMS JW, 2018, BMC PUBLIC HEALTH | POTENTIAL DRIVERS OF HIV ACQUISITION IN AFRICAN-AMERICAN WOMEN RELATED TO MASS INCARCERATION: AN AGENT-BASED MODELLING STUDY | Transmission Dynamics | Racial Disparities; Incarcerated | Philadelphia, PE - US | City | US | Yes | Yes |
| LUO W, 2018, JMIR PUBLIC HEALTH SURVEILL | DEVELOPMENT OF AN AGENT-BASED MODEL TO INVESTIGATE THE IMPACT OF HIV SELF-TESTING PROGRAMS ON MEN WHO HAVE SEX WITH MEN IN ATLANTA AND SEATTLE | Single Intervention | MSM | Atlanta, GA - US; Seatle, WA - US | City | US | No | Yes |
| GANTENBERG JR, 2018, PLOS ONE | IMPROVING THE IMPACT OF HIV PRE-EXPOSURE PROPHYLAXIS IMPLEMENTATION IN SMALL URBAN CENTERS AMONG MEN WHO HAVE SEX WITH MEN: AN AGENT-BASED MODELLING STUDY | PrEP | MSM | Rhode Island - US | State | US | No | Yes |
| GOEDEL WC, 2018, JAIDS | EFFECT OF RACIAL INEQUITIES IN PRE-EXPOSURE PROPHYLAXIS USE ON RACIAL DISPARITIES IN HIV INCIDENCE AMONG MEN WHO HAVE SEX WITH MEN: A MODELING STUDY | PrEP | MSM; Racial Disparities | Atlanta, GA - US | City | US | No | Yes |
| SCOTT N, 2018, J INT AIDS SOC | ELIMINATING HEPATITIS C VIRUS AS A PUBLIC HEALTH THREAT AMONG HIV-POSITIVE MEN WHO HAVE SEX WITH MEN: A MULTI-MODELLING APPROACH TO UNDERSTAND DIFFERENCES IN SEXUAL RISK BEHAVIOUR | Multidisease | MSM | Victoria - Australia | State | Australia | No | Yes |
| JOHNSON LF, 2018, EPIDEMIOL INFECT | AGE BIAS IN SURVEY SAMPLING AND IMPLICATIONS FOR ESTIMATING HIV PREVALENCE IN MEN WHO HAVE SEX WITH MEN: INSIGHTS FROM MATHEMATICAL MODELLING | Transmission Dynamics | MSM; Young | South Africa | Country | South Africa | No | Yes |
| LEVASSEUR MT, 2018, JAIDS | THE EFFECT OF PREP ON HIV INCIDENCE AMONG MEN WHO HAVE SEX WITH MEN IN THE CONTEXT OF CONDOM USE, TREATMENT AS PREVENTION, AND SEROADAPTIVE PRACTICES | Prevention Packages | MSM | US | Country | US | No | Yes |
| BERSHTEYN A, 2018, INFECT DIS MODEL | THE INFLUENCE OF MOBILITY AMONG HIGH-RISK POPULATIONS ON HIV TRANSMISSION IN WESTERN KENYA | Transmission Dynamics | FSW | Western Kenya | State | Kenya | No | Yes |
| MARSHALL BDL, 2018, LANCET HIV | POTENTIAL EFFECTIVENESS OF LONG-ACTING INJECTABLE PRE-EXPOSURE PROPHYLAXIS FOR HIV PREVENTION IN MEN WHO HAVE SEX WITH MEN: A MODELLING STUDY | PrEP | MSM | Atlanta, GA - US | City | US | No | Yes |
| WOOD D, 2018, JAIDS | RECRUITMENT OF FEMALE SEX WORKERS IN HIV PREVENTION TRIALS: CAN EFFICACY ENDPOINTS BE REACHED MORE EFFICIENTLY? | RCT Design | Women; FSW | South Africa | Country | South Africa | No | Yes |
| SHARMA M, 2018, AIDS | ASSISTED PARTNER NOTIFICATION SERVICES ARE COST-EFFECTIVE FOR DECREASING HIV BURDEN IN WESTERN KENYA | Single Intervention | Heterosexual | Nyanza, Kenya | State | Kenya | No | Yes |
| BASTEN M, 2018, AIDS | SEXUAL RISK BEHAVIOUR TRAJECTORIES AMONG MSM AT RISK FOR HIV IN AMSTERDAM, THE NETHERLANDS | Transmission Dynamics | MSM | Amsterdam, Netherlands | City | Netherlands | No | Yes |
| MCCREESH N, 2018, PLOS ONE | CHOICE OF TIME HORIZON CRITICAL IN ESTIMATING COSTS AND EFFECTS OF CHANGES TO HIV PROGRAMMES | Single Intervention | Representative | Uganda | Country | Uganda | No | Yes |
| CAMBIANO V, 2018, LANCET INFECT DIS | COST-EFFECTIVENESS OF PRE-EXPOSURE PROPHYLAXIS FOR HIV PREVENTION IN MEN WHO HAVE SEX WITH MEN IN THE UK: A MODELLING STUDY AND HEALTH ECONOMIC EVALUATION | PrEP | MSM | UK | Country | UK | No | Yes |
| ADAMS JW, 2019, PLOS ONE | DECREASING HIV TRANSMISSIONS TO AFRICAN AMERICAN WOMEN THROUGH INTERVENTIONS FOR MEN LIVING WITH HIV POST-INCARCERATION: AN AGENT-BASED MODELING STUDY | Prevention Packages | PWID; Racial Disparities; Incarcerated | Philadelphia, PE - US | City | US | No | Yes |
| MITTLER JE, 2019, PLOS COMPUT BIOL | LARGE BENEFITS TO YOUTH-FOCUSED HIV TREATMENT-AS-PREVENTION EFFORTS IN GENERALIZED HETEROSEXUAL POPULATIONS: AN AGENT-BASED SIMULATION MODEL | Single Intervention | Young | Sub-Saharan Africa | Macro-region | NA | No | No |
| CHAN PA, 2019, AIDS PATIENT CARE STDS | POTENTIAL IMPACT OF INTERVENTIONS TO ENHANCE RETENTION IN CARE DURING REAL-WORLD HIV PRE-EXPOSURE PROPHYLAXIS IMPLEMENTATION | PrEP | MSM | Rhode Island - US | State | US | No | Yes |
| SELINGER C, 2019, VACCINE | TARGETING AND VACCINE DURABILITY ARE KEY FOR POPULATION-LEVEL IMPACT AND COST-EFFECTIVENESS OF A POX-PROTEIN HIV VACCINE REGIMEN IN SOUTH AFRICA | Cost-Effectiveness | Heterosexual | South Africa | Country | South Africa | No | Yes |
| ISAAC AG, 2019, J ECON INTERACT COORD | PARTNERSHIP DURATION AND CONCURRENT PARTNERING: IMPLICATIONS FOR MODELS OF HIV PREVALENCE | Transmission Dynamics | Heterosexual | Sub-Saharan Africa | Macro-region | NA | No | No |
| OSETINSKY B, 2019, HEALTH AFF | EPIDEMIOLOGICAL AND HEALTH SYSTEMS IMPLICATIONS OF EVOLVING HIV AND HYPERTENSION IN SOUTH AFRICA AND KENYA | Multidisease | Representative | South Africa; Kenya | Country | South Africa; Kenya | No | Yes |
| REITSEMA M, 2019, AIDS | COST-EFFECTIVENESS OF INCREASED HIV TESTING AMONG MSM IN THE NETHERLANDS | Cost-Effectiveness | MSM | Netherlands | Country | Netherlands | No | Yes |
| BOBASHEV G, 2019, PLOS ONE | HEROIN TYPE, INJECTING BEHAVIOR, AND HIV TRANSMISSION. A SIMULATION MODEL OF HIV INCIDENCE AND PREVALENCE | Transmission Dynamics | PWID | US | Country | US | No | Yes |
| ANDERSSON E, 2019, EUROSURVEILLANCE | CHALLENGES IN MODELLING THE PROPORTION OF UNDIAGNOSED HIV INFECTIONS IN SWEDEN | Epidemiologic Analysis | Representative | Sweden | Country | Sweden | No | Yes |
| KASAIE P, 2019, BMJ OPEN | GONORRHOEA AND CHLAMYDIA DIAGNOSIS AS AN ENTRY POINT FOR HIV PRE-EXPOSURE PROPHYLAXIS: A MODELLING STUDY | PrEP | MSM | Baltimore City, Mariland - US | City | US | No | Yes |
| ABBAS UL, 2019, PLOS ONE | DRUG RESISTANCE FROM PREFERRED ANTIRETROVIRAL REGIMENS FOR HIV INFECTION IN SOUTH AFRICA: A MODELING STUDY | Proof of Concept | Heterosexual | Kwazulu-Natal, South Africa | State | South Africa | No | Yes |
| GOUNTAS I, 2019, J VIRAL HEPATITIS | A HEPATITIS C OUTBREAK PRECEDED THE HIV OUTBREAK AMONG PERSONS WHO INJECT DRUGS IN ATHENS, GREECE: INSIGHTS FROM A MATHEMATICAL MODELLING STUDY | Proof of Concept; Multidisease | PWID | Athens, Grece | City | Greece | NA | NA |
| PHILLIPS AN, 2019, J INT AIDS SOC | COST-PER-DIAGNOSIS AS A METRIC FOR MONITORING COST-EFFECTIVENESS OF HIV TESTING PROGRAMMES IN LOW-INCOME SETTINGS IN SOUTHERN AFRICA: HEALTH ECONOMIC AND MODELLING ANALYSIS | Cost-Effectiveness | Women; FSW; MSM; Heterosexual | NA | NA | NA | No | Yes |
| VERMEER W, 2020, JASSS | LEVERAGING MODULARITY DURING REPLICATION OF HIGH-FIDELITY MODELS: LESSONS FROM REPLICATING AN AGENT-BASED MODEL FOR HIV PREVENTION | Replication Exercise; Covid-19 | MSM | US | Country | US | Yes | No |
| GOEDEL WC, 2020, CLIN INFECT DIS | IMPLEMENTATION OF SYRINGE SERVICES PROGRAMS TO PREVENT RAPID HUMAN IMMUNODEFICIENCY VIRUS TRANSMISSION IN RURAL COUNTIES IN THE UNITED STATES: A MODELING STUDY | Single Intervention | PWID | Scott County, Indiana, US | City | US | No | Yes |
| SINGLETON AL, 2020, AIDS PATIENT CARE STDS | ADDED BENEFITS OF PRE-EXPOSURE PROPHYLAXIS USE ON HIV INCIDENCE WITH MINIMAL CHANGES IN EFFICIENCY IN THE CONTEXT OF HIGH TREATMENT ENGAGEMENT AMONG MEN WHO HAVE SEX WITH MEN | PrEP | MSM; Racial Disparities | Atlanta, GA - US; Seatle, WA - US | City | US | No | Yes |
| GOEDEL WC, 2020, AIDS | PROJECTING THE IMPACT OF EQUITY-BASED PREEXPOSURE PROPHYLAXIS IMPLEMENTATION ON RACIAL DISPARITIES IN HIV INCIDENCE AMONG MSM | PrEP | MSM; Racial Disparities | Atlanta, Sandy Springs, Alpharetta, GA - US | Micro-Region | US | No | Yes |
| GOEDEL WC, 2020, SCI REP | POTENTIAL IMPACT OF TARGETED HIV PRE-EXPOSURE PROPHYLAXIS UPTAKE AMONG MALE SEX WORKERS | PrEP | MSWS | Rhode Island - US | State | US | No | Yes |
| JOHNSON LF, 2020, PLOS ONE | CHALLENGES IN ESTIMATING HIV PREVALENCE TRENDS AND GEOGRAPHICAL VARIATION IN HIV PREVALENCE USING ANTENATAL DATA: INSIGHTS FROM MATHEMATICAL MODELLING | Proof of Concept | Women | South Africa | Country | South Africa | No | Yes |
| REITSEMA M, 2020, AIDS | PREEXPOSURE PROPHYLAXIS FOR MEN WHO HAVE SEX WITH MEN IN THE NETHERLANDS: IMPACT ON HIV AND NEISSERIA GONORRHOEAE TRANSMISSION AND COST-EFFECTIVENESS | Multidisease; PrEP; Transmission Dynamics; Cost-Effectiveness | MSM | Netherlands | Country | Netherlands | No | Yes |
| REITSEMA M, 2020, SEX TRANSM INFECT | IMPACT OF FREQUENT TESTING ON THE TRANSMISSION OF HIV ANDN. GONORRHOEAEAMONG MEN WHO HAVE SEX WITH MEN: A MATHEMATICAL MODELLING STUDY | Single Intervention | MSM | Netherlands | Country | Netherlands | No | Yes |
| KIM HY, 2020, J INT AIDS SOC | HIV SEROCONCORDANCE AMONG HETEROSEXUAL COUPLES IN RURAL KWAZULU-NATAL, SOUTH AFRICA: A POPULATION-BASED ANALYSIS | Epidemiologic Analysis | Heterosexual | Kwazulu-Natal, South Africa | State | South Africa | No | Yes |
| JENNESS SM, 2020, AIDS | MODELING AN INTEGRATED HIV PREVENTION AND CARE CONTINUUM TO ACHIEVE THE ENDING THE HIV EPIDEMIC GOALS | Prevention Packages | MSM | Atlanta, GA, US | City | US | No | Yes |
| XIAO X, 2020, J. Assoc. NURSES AIDS CARE | COGNITIVE IMPAIRMENT AMONG AGING PEOPLE LIVING WITH HIV ON ANTIRETROVIRAL THERAPY: A CROSS-SECTIONAL STUDY IN HUNAN, CHINA | Multidisease | MSM | Atlanta, Georgia, US | City | US | No | Yes |
| VERMEER W, 2020, J ARTIF SOC SOC SIMUL | LEVERAGING MODULARITY DURING REPLICATION OF HIGH-FIDELITY MODELS: LESSONS FROM REPLICATING AN AGENT-BASED MODEL FOR HIV PREVENTION. | Replication Exercise | MSM | Atlanta, GA - US | City | US | Yes | No |
| NIYUKURI D, 2021, MATHEMATICS | INFERRING HIV TRANSMISSION NETWORK DETERMINANTS USING AGENT-BASED MODELS CALIBRATED TO MULTI-DATA SOURCES | Transmission Dynamics | Heterosexual | Sub-Saharan Africa | Macro-region | NA | No | Yes |
| BUCHANAN AL, 2021, AM J EPIDEMIOL | DISSEMINATED EFFECTS IN AGENT-BASED MODELS: A POTENTIAL OUTCOMES FRAMEWORK AND APPLICATION TO INFORM PREEXPOSURE PROPHYLAXIS COVERAGE LEVELS FOR HIV PREVENTION | PrEP | MSM | Atlanta, GA - US | City | US | No | Yes |
| SINGH S, 2021, MATH BIOSCI ENG | PROGRESSION AND TRANSMISSION OF HIV (PATH 4.0)-A NEW AGENT-BASED EVOLVING NETWORK SIMULATION FOR MODELING HIV TRANSMISSION CLUSTERS | Transmission Dynamics | MSM; Heterosexual | US | Country | US | No | Yes |
| MCKAY VR, 2021, AIDS BEHAV | USING A MODELING-BASED APPROACH TO ASSESS AND OPTIMIZE HIV LINKAGE TO CARE SERVICES | LTC | Young | Memphis, TN - US | City | US | No | Yes |
| HENDRICKX DM, 2021, EPIDEMICS | INFLUENCE OF SEXUAL RISK BEHAVIOUR AND STI CO-INFECTION DYNAMICS ON THE EVOLUTION OF HIV SET POINT VIRAL LOAD IN MSM | Proof of Concept | MSM | Netherlands | Country | Netherlands | No | No |
| KHATAMI SN, 2021, MATH BIOSCI ENG | A REINFORCEMENT LEARNING MODEL TO INFORM OPTIMAL DECISION PATHS FOR HIV ELIMINATION | Proof of Concept | Representative | US | Country | US | No | Yes |
| KATZ DA, 2021, SEX TRANSM DIS | EFFECTS OF CONDOM USE ON HUMAN IMMUNODEFICIENCY VIRUS TRANSMISSION AMONG ADOLESCENT SEXUAL MINORITY MALES IN THE UNITED STATES: A MIXED EPIDEMIOLOGY AND EPIDEMIC MODELING STUDY | Single Intervention | YMSM | Delaware, Florida, Hawaii, Illinois, Michigan, New York City, Rhode Island, San Diego | Region | US | No | Yes |
| SINGLETON AL, 2021, EPIDEMICS | NETWORK STRUCTURE AND RAPID HIV TRANSMISSION AMONG PEOPLE WHO INJECT DRUGS: A SIMULATION-BASED ANALYSIS | Proof of Concept | PWID | Rural Indiana - US | Micro-Region | US | No | Yes |
| PEEBLES K, 2021, SCI REP | RISK COMPENSATION AFTER HIV-1 VACCINATION MAY ACCELERATE VIRAL ADAPTATION AND REDUCE COST-EFFECTIVENESS: A MODELING STUDY | Proof of Concept | Heterosexual | South Africa | Country | South Africa | No | Yes |
| BINGHAM A, 2021, SEX TRANSM DIS | ESTIMATED LIFETIME HIV-RELATED MEDICAL COSTS IN THE UNITED STATES | Cost-Effectiveness | PLWH | US | Country | US | No | Yes |
| GOYAL R, 2021, JAIDS | DEVELOPMENT OF A MATHEMATICAL MODEL TO ESTIMATE THE COST-EFFECTIVENESS OF HRSA'S RYAN WHITE HIV/AIDS PROGRAM | Cost-Effectiveness | MSM; PLWH | US | Country | US | No | Yes |
| KHANNA AS, 2021, MATH BIOSCI ENG | PROJECTING THE NUMBER OF NEW HIV INFECTIONS TO FORMULATE THE ``GETTING TO ZERO'' STRATEGY IN ILLINOIS, USA | Prevention Packages | Young; Racial Disparities; MSM | Illinois - US | State | US | No | Yes |
| GOYAL R, 2021, JAIDS-a | COST-EFFECTIVENESS OF HRSA'S RYAN WHITE HIV/AIDS PROGRAM? | Cost-Effectiveness | PLWH | US | Country | US | No | Yes |
| JEWELL BL, 2021, JAIDS | PREDICTING HIV INCIDENCE IN THE SEARCH TRIAL: A MATHEMATICAL MODELING STUDY | Epidemiologic Analysis | Heterosexual | Uganda; Kenya | Macro-region | Uganda; Kenya | No | Yes |
| SHARMA M, 2021, LANCET HIV | COST-EFFECTIVENESS OF POINT-OF-CARE TESTING WITH TASK-SHIFTING FOR HIV CARE IN SOUTH AFRICA: A MODELLING STUDY | Cost-Effectiveness | PLWH | Durban - South Africa | City | South Africa | NA | NA |
| THOMAS R, 2021, LANCET GLOB HEALTH | COST AND COST-EFFECTIVENESS OF A UNIVERSAL HIV TESTING AND TREATMENT INTERVENTION IN ZAMBIA AND SOUTH AFRICA: EVIDENCE AND PROJECTIONS FROM THE HPTN 071 (POPART) TRIAL | Cost-Effectiveness | Representative | Zambia; South Africa | Country | Zambia; South Africa | No | Yes |
| PHILLIPS AN, 2021, LANCET GLOB HEALTH | THE POTENTIAL ROLE OF LONG-ACTING INJECTABLE CABOTEGRAVIR-RILPIVIRINE IN THE TREATMENT OF HIV IN SUB-SAHARAN AFRICA: A MODELLING ANALYSIS | Cost-Effectiveness | PLWH | Sub-Saharan Africa | Macro-region | NA | No | Yes |
| JENNESS SM, 2021, J INFECT DIS | PROJECTED HIV AND BACTERIAL SEXUALLY TRANSMITTED INFECTION INCIDENCE FOLLOWING COVID-19-RELATED SEXUAL DISTANCING AND CLINICAL SERVICE INTERRUPTION. | Multidisease; Covid-19 | MSM | Atlanta, GA, US | City | US | No | Yes |
| BUCHANAN ALL, 2022, EPIDEMIOL INFECT | SPILLOVER BENEFIT OF PRE-EXPOSURE PROPHYLAXIS FOR HIV PREVENTION: EVALUATING THE IMPORTANCE OF EFFECT MODIFICATION USING AN AGENT-BASED MODEL | PrEP | MSM | Atlanta, Sandy Springs, Alpharetta, GA - US | Micro-Region | US | No | Yes |
| DEMEULEMEESTER R, 2022, BMC HEALTH SERV RES | ECONOMIC IMPACT OF GENERIC ANTIRETROVIRALS IN FRANCE FOR HIV PATIENTS' CARE: A SIMULATION BETWEEN 2019 AND 2023 | Cost-Effectiveness | PLWH | France | Country | France | Yes | Yes |
| VERMEER W, 2022, PLOS ONE | AGENT-BASED MODEL PROJECTIONS FOR REDUCING HIV INFECTION AMONG MSM: PREVENTION AND CARE PATHWAYS TO END THE HIV EPIDEMIC IN CHICAGO, ILLINOIS | Prevention Packages | MSM | Chicago, Illinois | City | US | Yes | Yes |
| LABS J, 2022, OPEN FORUM INFECT DIS | PROJECTED EFFECTS OF DISRUPTIONS TO HUMAN IMMUNODEFICIENCY VIRUS (HIV) PREVENTION SERVICES DURING THE CORONAVIRUS DISEASE 2019 PANDEMIC AMONG BLACK/AFRICAN AMERICAN MEN WHO HAVE SEX WITH MEN IN AN ENDING THE HIV EPIDEMIC PRIORITY JURISDICTION | Covid-19 | Racial Disparities | Mississippi | State | US | No | Yes |
| JACKA BP, 2022, JAIDS | BRIEF REPORT: USE OF PRE-EXPOSURE PROPHYLAXIS TO PREVENT RAPID HIV TRANSMISSION AMONG PEOPLE WHO INJECT DRUGS IN RURAL COUNTIES IN THE UNITED STATES: A MODELING STUDY | PrEP | PWID | Scott County, Indiana, US | City | US | NA | NA |
| NICHOLS BE, 2022, J INT AIDS SOC | FACILITY-BASED HIV SELF-TESTING STRATEGIES MAY SUBSTANTIALLY AND COST-EFFECTIVELY INCREASE THE NUMBER OF MEN AND YOUTH TESTED FOR HIV IN MALAWI: RESULTS FROM AN INDIVIDUAL-BASED MATHEMATICAL MODEL | Prevention Packages; Cost-Effectiveness | MSM; Young | Malawi | Country | Malawi | No | Yes |
| ZANG X, 2022, AIDS | THE IMPACT OF SYRINGE SERVICES PROGRAM CLOSURE ON THE RISK OF REBOUND HIV OUTBREAKS AMONG PEOPLE WHO INJECT DRUGS: A MODELING STUDY | Single Intervention | PWID | Scott County, Indiana, US | City | US | No | Yes |
| DES JARLAIS D, 2022, DRUG ALCOHOL DEPEND | MODELING HIV TRANSMISSION AMONG PERSONS WHO INJECT DRUGS (PWID) AT THE ``END OF THE HIV EPIDEMIC'' AND DURING THE COVID-19 PANDEMIC | Covid-19 | PWID | New York City, NY - US | City | US | No | Yes |
| MILWID RM, 2022, BMC INFECT DIS | PAST DYNAMICS OF HIV TRANSMISSION AMONG MEN WHO HAVE SEX WITH MEN IN MONTREAL, CANADA: A MATHEMATICAL MODELING STUDY | Transmission Dynamics | MSM | Montreal, Canada | City | Canada | No | Yes |
| KASAIE P, 2022, ANN EPIDEMIOL | PROJECTING THE AGE-DISTRIBUTION OF MEN WHO HAVE SEX WITH MEN RECEIVING HIV TREATMENT IN THE UNITED STATES | ART | MSM; PLWH | US | Country | US | No | Yes |
| ALTHOFF KN, 2022, AIDS | THE SHIFTING AGE DISTRIBUTION OF PEOPLE WITH HIV USING ANTIRETROVIRAL THERAPY IN THE UNITED STATES | ART | Representative | US | Country | US | No | Yes |
| HAMILTON DT, 2022, JAIDS | MODELING THE IMPACT OF HIV-1 NUCLEIC ACID TESTING AMONG SYMPTOMATIC ADULT OUTPATIENTS IN KENYA | Single Intervention | Representative | Kenya | Country | Kenya | No | Yes |
| BABIGUMIRA JB, 2022, BMJ OPEN | TESTING STRATEGIES TO DETECT ACUTE AND PREVALENT HIV INFECTION IN ADULT OUTPATIENTS SEEKING HEALTHCARE FOR SYMPTOMS COMPATIBLE WITH ACUTE HIV INFECTION IN KENYA: A COST-EFFECTIVENESS ANALYSIS | Cost-Effectiveness | Representative | Kenya | Country | Kenya | No | Yes |
| LEE F, 2022, INT J DRUG POLICY | STIMULANT USE INTERVENTIONS MAY STRENGTHEN `GETTING TO ZERO' HIV ELIMINATION INITIATIVES IN ILLINOIS: INSIGHTS FROM A MODELING STUDY | Transmission Dynamics | YBMSM; Racial Disparities | Illinois - US | State | US | No | Yes |
| ROBERTS DA, 2022, J INT AIDS SOC | THE IMPACT OF PREVENTION-EFFECTIVE PREP USE ON HIV INCIDENCE: A MATHEMATICAL MODELLING STUDY | PrEP; Cost-Effectiveness | Heterosexual | Eswantini | Country | Eswantini | No | Yes |
| CHEMAITELLY H, 2022, LANCET HIV | HIV INCIDENCE AND IMPACT OF INTERVENTIONS AMONG FEMALE SEX WORKERS AND THEIR CLIENTS IN THE MIDDLE EAST AND NORTH AFRICA: A MODELLING STUDY | Epidemiologic Analysis | FSW; Heterosexual | Algeria; Bahrain; Djibouti; Iran; Libya; Morocco; Pakistan; Somalia; South Sudan; Sudan; Tunisia; Yemen | Macro-region | NA | No | Yes |
| PROBERT WJM, 2022, Lancet HIV Health Educ. Behav. | PROJECTED OUTCOMES OF UNIVERSAL TESTING AND TREATMENT IN A GENERALISED HIV EPIDEMIC IN ZAMBIA AND SOUTH AFRICA (THE HPTN 071 [POPART] TRIAL): A MODELLING STUDY WHY CULTURE MATTERS IN HEALTH INTERVENTIONS: LESSONS FROM HIV/AIDS STIGMA AND NCDS | Proof of Concept; Transmission Dynamics | NA | Not Specified | NA | NA | No | No |
| ZHANG J, 2022, Int. J. Biomath. | DYNAMIC ANALYSIS OF AN HIV/AIDS TREATMENT MODEL INCORPORATING MSM | LTC | Representative | Zambia; South Africa | Macro-region | Zambia; South Africa | No | No |
| PROBERT WJM, 2022, LANCET HIV | PROJECTED OUTCOMES OF UNIVERSAL TESTING AND TREATMENT IN A GENERALISED HIV EPIDEMIC IN ZAMBIA AND SOUTH AFRICA (THE HPTN 071 [POPART] TRIAL): A MODELLING STUDY. | LTC | Representative | Zambia; South Africa | Macro-region | Zambia; South Africa | No | No |
| LEE F, 2023, MED CARE | EXPANDING MEDICAID TO REDUCE HUMAN IMMUNODEFICIENCY VIRUS TRANSMISSION IN HOUSTON, TEXAS INSIGHTS FROM A MODELING STUDY | PrEP | YBMSM; Racial Disparities | Houston, Texas, US | City | US | No | Yes |
| JACOBSON EU, 2023, AIDS CARE-PSYCHOL SOCIO-MED ASP AIDS-HIV | ASSESSING THE INDIVIDUAL BENEFITS OF REDUCING HIV DIAGNOSIS DELAY AND INCREASING ADHERENCE TO HIV CARE AND TREATMENT | Prevention Packages | PLWH | US | Country | US | NA | NA |
| KOENIG LJ, 2023, AIDS | CLOSING THE GAPS IN THE CONTINUUM OF DEPRESSION CARE FOR PERSONS WITH HIV: MODELING THE IMPACT ON VIRAL SUPPRESSION IN THE UNITED STATES | Single Intervention; Multidisease | PLWH | US | Country | US | No | Yes |
| HAMILTON DT, 2023, EPIDEMICS | MODELING THE IMPACT OF DIFFERENT PREP TARGETING STRATEGIES COMBINED WITH A CLINIC-BASED HIV-1 NUCLEIC ACID TESTING INTERVENTION IN KENYA | PrEP | Heterosexual | Kenya | Country | Kenya | No | Yes |
| MILALI MP, 2023, FRONT REPROD HEALTH | COST-EFFECTIVENESS OF THE DUAL PREVENTION PILL FOR CONTRACEPTION AND HIV PRE-EXPOSURE PROPHYLAXIS | Cost-Effectiveness | Women; Heterosexual | Kenya; Zimbabwe; South Africa | Macro-region | Kenya; Zimbabwe; South Africa | No | Yes |
| HAMILTON DT, 2023, BMC PUBLIC HEALTH | ACHIEVING THE ``ENDING THE HIV EPIDEMIC IN THE US'' INCIDENCE REDUCTION GOALS AMONG AT-RISK POPULATIONS IN THE SOUTH | Prevention Packages | MSM; Racial Disparities | South, US | Region | US | No | Yes |
| JOHNSON LF, 2023, BMC INFECT DIS | AN AGENT-BASED MODEL OF BINGE DRINKING, INEQUITABLE GENDER NORMS AND THEIR CONTRIBUTION TO HIV TRANSMISSION, WITH APPLICATION TO SOUTH AFRICA. | Epidemiologic Analysis | Heterosexual | South Africa | Country | South Africa | No | Yes |
| PICKLES M, 2023, LANCET GLOB HEALTH | STRENGTHENING THE HIV PREVENTION CASCADE TO MAXIMISE EPIDEMIOLOGICAL IMPACT IN EASTERN ZIMBABWE: A MODELLING STUDY. | LTC | Representative | Manicaland, Zimbabwe | State | Zimbabwe | No | No |

ODD: Overview, Design Concepts and Details protocol suggested by Grimm and colleagues for results’ presentation for Individual Based Methods (IBM). Limitations: If the article express, explicitly, its limitations. NA: Information that cannot be categorized or because of insufficient access to the document.
